# Ferruginous hemeprotein HhuH facilitates the cadmium adsorption and chromium reduction in *Stenotrophomonas* sp. SY1

**DOI:** 10.1128/aem.02097-24

**Published:** 2024-12-04

**Authors:** Zijie Zhou, Hongbo Yu, Jiahui Liu, Lin Zhu, Gejiao Wang, Kaixiang Shi

**Affiliations:** 1 National Key Laboratory of Agricultural Microbiology, College of Life Science and Technology, Huazhong Agricultural University 124443 , Wuhan, China; Georgia Institute of Technology, Atlanta, Georgia, USA

**Keywords:** cadmium adsorption, chromium reduction, iron starvation, hemeprotein

## Abstract

Cadmium (Cd) and chromium (Cr) are frequently encountered toxicants, while iron (Fe) plays a crucial role in bacterial survival under conditions of heavy metal stress. However, intracellular Fe ion depletion by heavy metals leads to a state of Fe starvation. Therefore, it is imperative to investigate the mechanism through which bacteria maintain a balance between heavy metal detoxification and Fe homeostasis. This study demonstrates Cd(II) immobilization and Cr(VI) reduction abilities of *Stenotrophomonas* sp. SY1, while proteomics reveals the upregulation of heme metabolism in response to Cd(II) and Cr(VI) exposure. The expression of the heme-uptake system in *Escherichia coli* can enhance Cd(II) immobilization and facilitate Cr(VI) reduction. The ferruginous hemeprotein HhuH exhibits the ability to chelate Cd(II) and reduce Cr(VI). The presence of Cd(II) and Cr(VI) in strain SY1 initially led to Fe starvation. Subsequently, the hemeprotein HhuH facilitated Cd(II) adsorption and Cr(VI) reduction, thereby restoring normal cellular Fe homeostasis. Our findings explain the hemeprotein-mediated mechanism for Cd(II) adsorption and Cr(VI) reduction, providing further insights into the correlation between heavy metal and Fe metabolism.

**IMPORTANCE:**

Iron (Fe) is an indispensable trace element for many organisms, and virtually, all bacteria require Fe as a cofactor in enzymes to facilitate redox reactions involved in fundamental cellular processes during periods of heavy metal stress. Understanding bacterial response to Fe in heavy metal contamination is essential. Therefore, our study elucidates Cd(II) adsorption and Cr(VI) reduction processes mediated by the Fe-bearing hemeprotein HhuH. It is a unique trifunctional protein capable of chelating Cd(II) and reducing Cr(VI), demonstrating significant potential in the environmental remediation of heavy metals.

## INTRODUCTION

Cadmium (Cd) and chromium (Cr) are two commonly found toxic heavy metals (1). Cd and Cr are classified as priority pollutants by the United States Environmental Protection Agency and the European Union (2, 3). In nature, Cd primarily exists as a divalent ion Cd(II), while Cr can exist in two main valence states: the low-toxic trivalent ion Cr(III) and the highly toxic hexavalent ion Cr(VI) (4). In the environment, Cd(II) and Cr(VI) can enter the body through ingestion, inhalation, and dermal exposure (5–7). When living in areas with Cd(II) and Cr(VI) pollution, humans are at a significant risk of poisoning (5–7). Cd(II) and Cr(VI) are non-threshold toxicants, exerting harmful effects even at low concentrations (8). Cd(II) causes damage to various organs, including the brain, kidneys, bones, lungs, liver, and reproductive system (9, 10), whereas Cr(VI) adversely affects multiple systems such as respiratory, cardiovascular, digestive, immune, nervous, and reproductive systems (11). The reduction of environmental Cd(II) and Cr(VI) hazards is crucial for human well-being.

The bacteria-mediated Cd(II) adsorption and Cr(VI) reduction are effective methods for remediating pollution caused by Cd(II) and Cr(VI), respectively (1). Iron (Fe) plays a crucial role in these processes (12, 13). Sulfate-reducing bacteria produce Fe(II) minerals that can adsorb Cd(II) or reduce Cr(VI) to Cr(III) (12, 13). The depletion of intracellular Fe ions, known as Fe starvation, is a detrimental effect of heavy metal toxicity. During Fe uptake, extracellular heavy metals will compete with Fe for entry into cells through the Fe-uptake transporter, thereby reducing its uptake (14–17). Intracellular heavy metals can activate cation diffusion facilitators (efflux transporters of multiple ions such as Cd and Fe), increasing Fe efflux (18). Consequently, bacteria exposed to heavy metal stress often undergo a state of Fe starvation (19, 20). The limited availability of Fe poses a challenge in meeting the requirements of all Fe-bearing proteins (15). In response to Fe starvation, bacteria downregulate the expression of numerous Fe-bearing proteins to conserve Fe consumption (15). Interestingly, heme Fe consumption is preferred during Fe starvation caused by heavy metals (15). Nevertheless, further investigation is required to elucidate the underlying mechanism behind this bacterial preference for heme in heavy metal-induced Fe starvation.

The *Stenotrophomonas* is a widely distributed environmental microorganism, particularly found in soil and plants (21). This genus plays a crucial role as a beneficial microorganism for plants and finds extensive applications in agriculture (21). In terms of pollution remediation, *Stenotrophomonas* exhibits the ability to degrade various environmental pollutants such as phenols (22), polycyclic aromatic hydrocarbons (23), and selenium compounds (24). Additionally, this genus demonstrates remarkable adsorption capabilities toward multiple heavy metals, thus showcasing its significant potential in remediating heavy metal pollution (25–27). In plant growth promotion, *Stenotrophomonas* is capable of producing the plant growth hormone indole-3-acetic acid (28). Moreover, this genus plays a crucial role in the nitrogen and sulfur cycles, thereby contributing to plant growth (21). In terms of biocontrol properties, the genus *Stenotrophomonas* exhibits effective antibacterial activity against plant pathogenic fungi through the secretion of chitinases, maltophilin, and xanthobaccin (21). Fe is an essential nutrient for *Stenotrophomonas*, playing a critical role in biofilm formation, oxidative stress response, and expression of outer membrane proteins (29, 30). Two distinct pathways, namely heme-mediated and siderophore-mediated pathways, are involved in the uptake of Fe by *Stenotrophomonas* (30). In the siderophore-mediated Fe uptake system, this genus utilizes multiple siderophore (stenobactin, pyochelin, and pseudobactin) to acquire Fe from the environment (31–33). Both genotypic and phenotypic investigations have reported the presence of a heme-mediated Fe acquisition system in *Stenotrophomonas*, which includes heme oxygenase, heme ATP-binding cassette (ABC) transporter protein complex, as well as hemin uptake and transport proteins (30). During Fe starvation conditions in *Stenotrophomonas*, heme is utilized as an iron source to sustain growth (29).

The strain *Stenotrophomonas* sp. SY1 was isolated from the soil of a pig farm (34). In this study, strain SY1 demonstrated efficient adsorption of Cd(II) and reduction of Cr(VI), with the addition of Fe to the cultivation medium further enhancing these abilities. Proteomics analysis unveiled the presence of a heavy metal-induced specific heme-uptake gene cluster *hhu*, which includes a hemeprotein gene (we have designated as *hhuH*) and four heme-uptake transporter genes (*fepA*, *chuT*, *fecCD*, and *hmuV*) (35–38). Subsequent experiments confirmed that the hemeprotein HhuH can chelate Cd(II) and reduce Cr(VI). Solving heavy metal stress can restore normal Fe homeostasis in cells. Our findings explain the specific mechanism by which bacteria adsorb Cd(II) and reduce Cr(VI), mediated by hemeprotein, thereby providing further insights into the correlation between heavy metal and Fe metabolism.

## MATERIALS AND METHODS

### Bacteria, culture media, chemical agents, and plasmid

*Stenotrophomonas* SY1 was isolated from the soil of a pig farm in Shayang County, Jingmen City, Hubei Province, China, as reported in a previous study (34). The strain was cultivated using Luria-Bertani (LB) medium and stored with 25% glycerol at −80°C. LB medium was prepared with 10 g/L tryptone, 5 g/L yeast extract, and 10 g/L sodium chloride. The 0.1 M Cd(II), 1 M Cr(VI), and 0.5 M Fe(III) stock solutions were prepared by dissolving cadmium chloride (CdCl_2_), potassium chromate (K_2_CrO_4_), and ferric chloride (FeCl_3_) in water, respectively. The stock solutions were stored in sealed containers at room temperature. The plasmids used in this study are listed in Table 1.

**TABLE 1:** Plasmids used in this study

Plasmid	Description	Source
pCT-Zori	Expression plasmid; Cm^r^	(39)
pCT-Zori-*hhu*	pCT-Zori vector cloned with whole *hhu* gene cluster	This study
pET28a	His-Tag overexpression vector, Km^r^	Novagen
pET28a-*hhuH*	The overexpression vector of HhuH	This study

### Minimal inhibitory concentration assays

To investigate the tolerance of stain SY1 toward Cd(II) or Cr(VI), a 250 mL conical flask with a capacity of 100 mL fresh LB medium was inoculated with 1 mL bacterial solution of strain SY1 (OD_600_ ≈ 1.0). The LB medium was supplemented with either 0 or 50 µM FeCl_3_. Subsequently, different concentrations of Cd(II) (0.05, 0.1, 0.2, 0.3, 0.4, or 0.5 mM) or Cr(VI) (0.1, 0.2, 0.5, 1, 2, 3, or 5 mM) were added to these media. Strain SY1 was then cultivated at a temperature of 28°C and an agitation speed of 150 rpm. After incubation for 48 h, the OD_600_ of the bacterial solution was measured using a UV-visible spectrophotometer (UV1900; Shanghai AOE Instruments Co., Ltd., China).

### Atomic absorption spectrophotometry

The 1 mL bacteria solution of strain SY1 (OD_600_ ≈ 1.0) was cultivated in 250 mL capacity conical flask with 100 mL of fresh LB medium containing no heavy metals, 50 µM Cd(II), 100 µM Cr(VI), or 50 µM Cd(II) + 100 µM Cr(VI) at 28°C and 150 rpm. Subsequently, the bacterial solutions were collected at 12, 20, 24, 36, and 48 h time point. Afterward, the bacterial solution was centrifuged at room temperature for 5 min at 12,000 rpm to separate cell and supernatant to measure total Cr and Cd. Finally, cell was subjected to digestion with 50% nitric acid at 70°C until the solution became completely clear. The concentrations of Cd and Cr were determined using an atomic absorption spectrophotometry (AAS) technique in this study. The measurements were performed on a TAS-990 atomic absorption spectrophotometer (Persee General Instrument Co., Ltd., Beijing, China). The detection limits for this instrument were 0.0164 µM for Cd and 0.148 µM for Cr. The concentrations were calculated based on the equation: the measured concentration × dilution rate × total bacterial solution volume (100 mL). The Cd(II) adsorption of strain SY1 was equal to the total Cd content in the cell.

To explore the impact of Fe on bacterial Cd(II) adsorption and Cr(VI) reduction, 1 mL bacteria solution of strain SY1 was added in 250 mL capacity conical flask with 100 mL of LB medium containing 50 µM FeCl_3_, 50 µM FeCl_3_ + 50 µM Cd(II), 50 µM FeCl_3_ + 100 µM Cr(VI), or 50 µM FeCl_3_ + 50 µM Cd(II) + 100 µM Cr(VI). The cultivation process was performed at 28°C and 150 rpm. Subsequently, the bacterial solution was collected and analyzed as described previously.

### Colorimetric diphenylcarbazide

The diphenylcarbazide (DPC) method was employed for the detection of Cr(VI) in this study. In the DPC method, diphenylcarbazide can form a complex with Cr(VI), resulting in a characteristic absorption peak at 540 nm (38). The diphenylcarbazide color reagent was prepared by dissolving 200 mg of diphenylcarbazide in 100 mL of 50% acetone. For each diluted sample, 200 µL of H_2_O, 10 µL of 50% sulfuric acid, 10 µL of 50% phosphoric acid, and 40 µL of diphenylcarbazide color reagent were sequentially added. After thorough mixing, the mixture was allowed to stand for 10 min. The absorbance at OD_540_ was measured using a BioTek Cytation5 microplate reader (BioTek Instruments Co., Ltd., Vermont, USA). The obtained OD_540_ value was used to calculate the concentration of Cr(VI). To measure the Cr(VI) reduction by strain SY1, the supernatant of bacteria solution was obtained as described previously. The Cr(VI) content in supernatant was detected by DPC. The Cr(VI) reduction was equal to the difference value of total Cr content and Cr(VI) content in supernatant.

### Quantitative reverse transcription PCR

In this study, quantitative reverse transcription PCR (RT-qPCR) was employed to quantify the transcription levels of the key gene *hhuH*. A 1 mL bacteria solution of strain SY1 (OD_600_ ≈ 1.0) was inoculated into a 250 mL conical flask containing 100 mL of fresh LB medium with either no heavy metals, 50 µM Fe(III), or supplemented with 50 µM Cd(II), 100 µM Cr(VI), or 50 µM Cd(II) + 100 µM Cr(VI). The cultures were then incubated at 28°C and 150 rpm. Subsequently, bacterial solutions were collected at time points of 12, 20, 24, 36, and 48 h. Cells were separated from the bacterial solutions through centrifugation (at room temperature for 5 min at 12,000 rpm) for RT**-**qPCR analysis. The total nucleic acid (including DNA and RNA) of these cells was extracted using TRIzol (Servicebio Technology Co., Ltd., Wuhan, China), followed by the removal of genomic DNA from the extracted nucleic acid using genomic DNA (gDNA) wiper Mix (Vazyme Biotech Co., Ltd., Nanjing, China). Subsequently, cDNA was synthesized through reverse transcription with HiScript II qRT SuperMix II^a^ (Vazyme Biotech Co., Ltd., Nanjing, China). Finally, the obtained cDNA was quantified by RT-qPCR using AceQ qPCR SYBR Green Master Mix (Vazyme Biotech Co., Ltd., Nanjing, China). The annealing temperature was 60°C, and cycles number was 40 times. The primers for the target gene *hhuH* were hhuH-F: CCCGGCCAGAAGGTGAC and hhuH-R: GCACTGGTCACGAACGC. The primers for the internal control 16S rRNA gene were 16 S-F: GGCAGCACAGGAGAGCTT and 16 S-R: CCGTAGGAGTCTGGACCG. The amplification efficiency for primers hhuH-F/hhuH-R and 16S-F/16S-R was 106.86% and 113.86%, respectively. The RT-qPCR instrument used was Applied Biosystems QuantStudio 3 (Thermo Fisher Scientific, Waltham, MA, USA).

### Isobaric tags for relative and absolute quantitation proteomics

To prepare the proteomics samples, the 1 mL bacteria solution of strain SY1 (OD_600_ ≈ 1.0) was cultivated in a 250 mL conical flask containing 100 mL of fresh LB medium, either without the addition of heavy metals or supplemented with 50 µM Cd(II) + 100 µM Cr(VI) at 28°C and 150 rpm. The cells were harvested at the 20 h time point through centrifugation (8,000 rpm at 4°C for 10 min) and subsequently subjected to proteomics analysis using isobaric tags for relative and absolute quantitation labeling/fractionation and liquid chromatography coupled to tandem mass spectrometry (LC–MS/MS) techniques provided by Wuhan Gene Create Ltd. (Wuhan, China). According to the method description provided by Wuhan Gene Create Ltd. (Wuhan, China), the genome sequence JAJOYR010000001 (from NCBI database) was used for peptide library generation. Subsequently, the mass spectrometry proteomics data were analyzed using Proteopilot 4.5 software with this peptide library. The one-sample *t*-test was used to compare relative abundance. In cases where the *P*-value (*t*-test) was ≤0.05, significant change in abundance was defined as fold changes ≥1.5 or ≤−1.5 between different groups.

### Heterologous expression of the heme-uptake gene cluster

The heme-uptake gene cluster *hhu* was heterologously expressed in *Escherichia coli* DH5α, which lacks a similar gene cluster, to investigate its roles. For this experiment, the expression plasmid pCT-zori was selected, containing a Lac promoter and *pUC* replication origin (with a copy number of 257 in *E. coli*) (39, 40). The detailed plasmid profile of pCT-zori is provided in Fig. S1. We amplified the gene cluster *hhu* using PCR with the primers *hhu*-F 5ʹ-CGACTCTAGAGGATCCACCTTGTATCGGCTGGTG-3ʹ and *hhu*-R 5ʹ-TCGGTACCCGGGGATCCTCATTCGTCGCAAGGCAC-3ʹ. The annealing temperatures of this PCR were 56°C. The plasmid pCT-Zori was digested with the restriction enzyme *Bam*HI. The resultant PCR fragments were purified and ligated into the plasmid pCT-Zori through homologous recombinase. Subsequently, these recombinant plasmids were transformed into *E. coli* DH5α, and the transformants were screened out by LB medium plates containing 50 µg/mL of chloramphenicol. To identify correctly expressed heterologous strains, these transformants were verified by PCR (using common primers M13F/M13R) followed by DNA sequencing.

The 1 mL bacteria solution (strain DH5α-pCT-Zori or DH5α-pCT-Zori-*hhu*) was cultivated in a 250 mL conical flask with 100 mL of fresh LB medium containing 50 µM Cd(II), 100 µM Cr(VI), or 50 µM Cd(II) + 100 µM Cr(VI) at 37°C and 150 rpm. Subsequently, a sample of the bacterial solution (5 mL) was taken at time points of 12, 20, 24, and 36 h. The cells were then separated from the supernatant by centrifugation at room temperature for 5 min at 6,000 rpm. Afterward, the cells were resuspended in 1.5 mL of 10 mM Tris-HCl buffer (pH 8.0) containing 0.1 mM *N,N*'-dicyclohexylcarbodiimide, an inhibitor for bacterial efflux pump (41), and 0.6 mL chloroform was added to release periplasmic contents. The mixture was incubated at room temperature for 15 min after brief vortexing, followed by the addition of 5 mL of 10 mM Tris-HCl buffer (pH 8.0). Subsequently, this mixture was centrifuged at room temperature and 6,000 rpm for 5 min to separate the periplasm (dissolved in supernatant) from the cytoplasm (present in sediment). Finally, the sediment containing cytoplasm was digested with 50% nitric acid at 70°C until complete clarity of the digestion solution was achieved. AAS was employed to measure total Cd and Cr in the supernatant sample, while DPC was utilized for Cr(VI) detection.

### Purification of HhuH

To purify the hemeprotein HhuH *in vitro*, the target gene *hhuH* was amplified by PCR using the primers *hhuH*-F: 5ʹ-AAAGAATTCTTACTGCTTCGGTACGAAAC-3ʹ and *hhuH*-R: 5ʹ-AAAGGATCCATGACGGTCATTCGTTCC-3ʹ. The PCR annealing temperature was 54°C. The purified PCR amplification product and plasmid pET28a were subjected to digestion using the restriction enzymes *Eco*RI and *Bam*HI. Subsequently, the digested target gene was ligated into plasmid pET28a using T4 ligase, thereby constructing a recombinant plasmid for HhuH overexpression. The recombinant plasmid was transformed into *E. coli* BL21, and the transformants were screened on LB medium plates containing 50 µg/mL of kanamycin. To identify correct *E. coli* BL21-pET28a-*hhuH* from the above transformants, these transformants were verified using PCR and subjected to DNA sequencing.

Five milliliter of *E. coli* BL21-pET28a-*hhuH* strain was inoculated into 500 mL of LB medium and incubated at 37°C with 150 rpm. Once the OD_600_ reached 0.3–0.4, isopropyl β-D-thiogalactoside (IPTG) was added at a final concentration of 1 mM to induce protein expression, followed by further incubation at 28°C and 150 rpm for 12 h. Subsequently, cells were harvested by centrifugation (8,000 rpm at 4°C for 5 min) and resuspended in a total volume of 15 mL of phosphate buffer (0.05 M, pH 7.0). Furthermore, the bacterial suspension was lysed using a low-temperature ultrahigh-pressure continuous-flow cell disrupter (JN3000PLUS, Juneng Nano and Bio-Technology Co., Ltd., Guangzhou, China) operating at 1,000 MPa. The supernatant containing the lysate was collected by centrifugation (8,000 rpm at 4°C for 15 min).

HhuH was purified using an affinity column containing Ni NTA beads (Smart-Lifesciences Biotechnology Co., Ltd., Changzhou, China). The supernatant of the lysate was incubated in the affinity column at 4°C for 30 min. Subsequently, the affinity column was washed sequentially with 20 mL of phosphate buffer (0.05 M, pH 7.0) and 15 mL of phosphate buffer (0.05 M, pH 7.0) containing 30 mM of imidazole. Afterward, the protein HhuH was eluted from the affinity column using 15 mL phosphate buffer (0.05 M, pH 7.0) containing 500 mM imidazole. The eluted HhuH was dialyzed with a phosphate buffer (0.05 M, pH 7.0) to remove residual imidazole. Finally, the purified HhuH was subjected to analysis by SDS-PAGE (42) and LC-MS (provided with Wuhan Gene Create Ltd., Wuhan, China). The absorption peak corresponding to the HhuH protein was detected using an UV-visible spectrophotometer (UV1900, Shanghai AOE Instruments Co., Ltd, China). The heme of HhuH protein was verified using a colorimetric measurement with 2,2′-azinobis [3-ethylbenzothiazoline-6-sulfonic acid]-diammonium salt (ABTS), performed using the Hemeprotein Colorimetric Assay Kit (Beyotime Biotechnology Co., Ltd., Shanghai, China). Bovine serum albumin and standard hemoglobin were employed as negative and positive controls, respectively, in the ABTS measurement. The purified protein was subjected to digestion with 50% nitric acid at 70°C for 1 h to determine its Fe content using AAS.

### Protein localization of HhuH

To analyze the subcellular localization of HhuH, a 5 mL of bacteria solution (the strain BL21-pET28a-*hhuH*) was cultivated in 500 mL fresh LB medium at 37°C with 150 rpm. Subsequently, IPTG was added to a final concentration of 1 mM to induce protein expression until the OD_600_ reached 0.3–0.4, and further cultivation was carried out at 28°C with 150 rpm for 12 h. Then, cells were harvested by centrifugation (8,000 rpm at room temperature for 5 min) and washed twice with buffer A (75 mM of Hepes-KOH, 15 mM of KCl, 1 mM of MgSO_4_, 250 mM of sucrose, and pH = 7.5) in a volume of 100 mL each time. The washed cells were resuspended in buffer A containing DNase I (100 µg) and incubated at room temperature for 20 min. Finally, the resuspended solution was supplemented sequentially with MgSO_4_ (4 mM), dithiothreitol (2 mM), and phenylmethylsulfonyl fluoride (1 mM). The mixture was lysed using a low-temperature ultrahigh-pressure continuous-flow cell disrupter (JN3000PLUS, Juneng Nano and Bio-Technology Co., Ltd., Guangzhou, China) operating at 1,000 MPa. The supernatant, containing cytoplasm and cytomembrane, and the sediment, containing cell fragments, were separated by centrifugation (8,000 rpm at 4°C for 15 min). Subsequently, the cytoplasm and cytomembrane were further separated through ultracentrifugation (150,000 g at 4°C for 1 h). Following ultracentrifugation, the supernatant contained the cytoplasm, while the sediment contained the cytomembrane. The HhuH protein was detected in various components, including the supernatant of the bacteria solution (containing cell secretions), sediment of lysate (containing cell fragments), supernatant after ultracentrifugation (containing cytoplasm), and sediment after ultracentrifugation (containing cytomembrane) using an enzyme linked immunosorbent assay (ELISA). All reagents for ELISA were provided by Beyotime Biotechnology Co., Ltd, Shanghai, China. The experimental procedure for the ELISA was referenced from Zhou et al. (43).

### Site-directed mutagenesis of hemeprotein HhuH

The mutant protein was generated through site-directed mutagenesis, wherein alanine was substituted for R127 of HhuH. A mutational vector was amplified from the template plasmid pET28a-*hhuH* using extension PCR with the following primers: R127A-F: 5ʹ-GCACATCCGCCGAGACCTTCAGCTCGAAGA-3ʹ and R127A-R: 5ʹ-AAGGTCTCGGCGGATGTGCCGCTGTCCGGGC-3ʹ. The annealing temperatures for this PCR were set at 60°C. Subsequently, the PCR products (including both intact mutational plasmid and template plasmid) were purified and digested with the restriction enzyme *Dpn*I at 37°C for 1 h to remove the template plasmid. The digestion reaction was terminated at 65°C for 10 min. Subsequently, the resulting digested product was directly transferred into *E. coli* BL21 cells for sequence confirmation (44). The purification method for obtaining the mutant protein R127A was similar to that used for expressing and purifying wild-type HhuH.

### Interaction experiment between hemeprotein HhuH and Cd(II)

The purified HhuH protein (19 µM) and Cd(II) (100 µM) were added to a 10 mL system containing phosphate buffer (0.05 M, pH 7.0). Subsequently, the mixture was incubated at room temperature for 30 min. Afterward, the Cd-HhuH complex was purified from the mixture using the same purification method as that for wild-type HhuH. The absorption peak of Cd-HhuH was detected using a UV-visible spectrophotometer. To eliminate the influence of the His-tag on Cd binding, enzymatic excision of the His-tag from Cd-HhuH was performed using thrombin (Beyotime Biotechnology Co., Ltd, Shanghai, China). Following Ni affinity column capture of Cd-HhuH from the HhuH-Cd(II) interaction system, thrombin was added to the Ni affinity column for overnight enzymatic excision at 4°C, allowing for elution of Cd-HhuH without the His-tag. Subsequently, Cd-HhuH without the His-tag was digested with 50% nitric acid at 70°C for 1 h and analyzed for Fe and Cd content by AAS. The AAS data are presented in Table S1.

### Molecular docking of hemeprotein HhuH

To predict the interaction site of HhuH and Cr(VI), the structural model of HhuH was generated through homology modeling using SWISS-Model (45), while that of Cr(VI) was directly obtained from PubChem databases (https://pubchem.ncbi.nlm.nih.gov). The interaction of HhuH and Cr(VI) was predicted by molecular docking using AutoDock 4.2 (46). The docked complex was visualized using PyMOL v2.5 (47). The 3D structures of wild-type HhuH and mutant R127A were compared using PyMOL v2.5, and the root mean squared deviations (RMSDs) between the two proteins were utilized to quantify their differences in terms of 3D structure (47).

### Cr(VI) reduction of HhuH

For the investigation of the impact of different electron donors (NADH and NADPH) on the enzymatic activity of HhuH, 10 µg/mL of HhuH was incubated with 5 mM of either NADH or NADPH and varying concentrations (0.1, 0.2, 0.4, 0.6, 1, 2, 3, and 4 mM) of Cr(VI) for 15 min at room temperature in a reaction volume of 100 µL phosphate buffer (0.05 M, pH 7.0). The remaining Cr(VI) concentration was determined using the DPC analysis method (48). The presence of Cr(VI) at 340 nm (Fig. S2) interfered with the accurate detection of NADH or NADPH oxidation rates. Therefore, the rate of Cr(VI) consumption was utilized as a representative measure of enzymatic activity. The equation (1) was employed to calculate the Cr(VI) reductase activity.


(1) $$Enzymatic\;activity\;\left(\mu mol/min/mg\right)=\frac{C_{0}-C_{t}}{t\times C_{P}}$$

There *t* (min) was the reduction reaction time, *C_0_* (μM) was the initial concentrations of substrate, *C_t_* (μM) was the residual concentrations of substrate after time *t*, and *C_p_* (mg/L) was the concentrations of hemeprotein. GraphPad Prism software version 9.5 was employed to analyze the reduction characteristics of Cr(VI) by HhuH. In the non-enzyme negative control experiment, a mixture of 5 mM NADH or NADPH and 1 mM Cr(VI) were prepared in 100 µL phosphate buffer (0.05 M, pH 7.0) and allowed to react for 15 min at room temperature. The background activity experiment of HhuH was performed by incubating 10 µg/mL HhuH and 1 mM Cr(VI) in 100 µL phosphate buffer (0.05 M, pH 7.0) for a duration of 15 min at room temperature.

To investigate the difference in enzymatic activity between wild-type HhuH, R127A, and Cd-HhuH variants, each protein variant (10 µg/mL) was separately incubated with an electron donor NADH (5 mM), along with 1 mM Cr(VI) for 15 min in a 100 µL reaction volume at room temperature in phosphate buffer (0.05 M, pH 7.0). To conduct the enzyme inhibition assay, 10 mM of the metal-chelator EDTA was introduced as an inhibitor for HhuH into a reaction system consisting of 10 µg/mL of HhuH, 5 mM of NADH, and 1 mM of Cr(VI) in a 100 µL volume of phosphate buffer (0.05 M, pH 7.0). The reaction was carried at room temperature for 15 min.

### Nitrite reduction of HhuH

The nitrite reduction of HhuH was assessed using the method described by Zhang et al (49). A freshly prepared solution of 0.1 M sodium hydrosulfite (Na_2_S_2_O_4_) in 0.1 M NH_4_HCO_3_ was used. The reaction system for nitrite reduction consisted of 10 µg/mL of HhuH, 0.5 mM of methyl viologen, 5 mM Na_2_S_2_O_4_, phosphate buffer (0.05 M, pH 7.0), and varying concentrations (0.1, 0.2, 0.4, 0.6, 1, 2, 3, and 4 mM) of NaNO_2_ in a total volume of 100 µL. The reaction was initiated by adding Na_2_S_2_O_4_ and allowed to proceed for 15 min at room temperature before being terminated by vigorous agitation. The remaining nitrite in the reaction system was quantified using diazonium coupling reaction (49). In this study, the samples were pre-diluted based on a standard curve. Subsequently, a mixture of 200 µL H_2_O, 50 µL diluted sample, and 40 µL solution of aminobenzene sulfonic acid (4 g/L) was prepared in a tube and allowed to stand for 5 min. Then, 20 µL solution of N-(1-naphthyl) ethylenediamine dihydrochloride (2 g/L) from the previous step was added to the mixture and allowed to stand for additional 15 min. Finally, the OD_538_ of the resulting mixture was measured using a BioTek Cytation5 microplate reader (BioTek Instruments Co., Ltd., Vermont, USA). The OD_538_ value was used to calculate the remaining concentration of NaNO_2_ according to equation (1). The consumption rate of NaNO_2_ in this process represented nitrite reductase activity.

### Analysis of phylogenetic tree and conservative motifs

The taxonomic information of HhuH was obtained by analyzing its phylogenetic tree and conservative motifs. The neighbor-joining method in MEGA 6.0 was used to construct the phylogenetic tree of HhuH along with other hemeproteins and Cr(VI) reductases (49). The resulting tree was visualized using FigTree v1.4.3 (http://tree.bio.ed.ac.uk/software/figtree/). To differentiate hemeprotein HhuH from its homologs, their conserved motifs were predicted using MEME (https://meme-suite.org/meme/tools/meme).

## RESULTS

### *Stenotrophomonas* sp. SY1 exhibited ability to adsorb Cd(II) and reduce Cr(VI) efficiently

The *Stenotrophomonas* sp. SY1 strain exhibited high tolerance to heavy metals, with MIC values of 300 µM for Cd(II) and 2000 µM for Cr(VI) in LB medium cultivation (Fig. S3). The background concentrations of Cd and Cr in the LB medium were measured as 3.37 and 6.07 µM, respectively. The LB medium exhibits no reactivity toward Cd(II) and Cr(VI), while the addition of Fe(III) supplement in LB medium does not induce any reaction with Cd(II) or Cr(VI) (Fig. S4). Considering that excessively high concentrations of heavy metals significantly inhibit the growth of strain SY1, while much lower concentrations are insufficient to demonstrate its ability in Cd(II) adsorption and Cr(VI) reduction aspects, the strain SY1 was cultivated in LB medium containing 50 µM Cd(II), 100 µM Cr(VI), or 50 µM Cd(II) + 100 µM Cr(VI).

**Fig 1:**
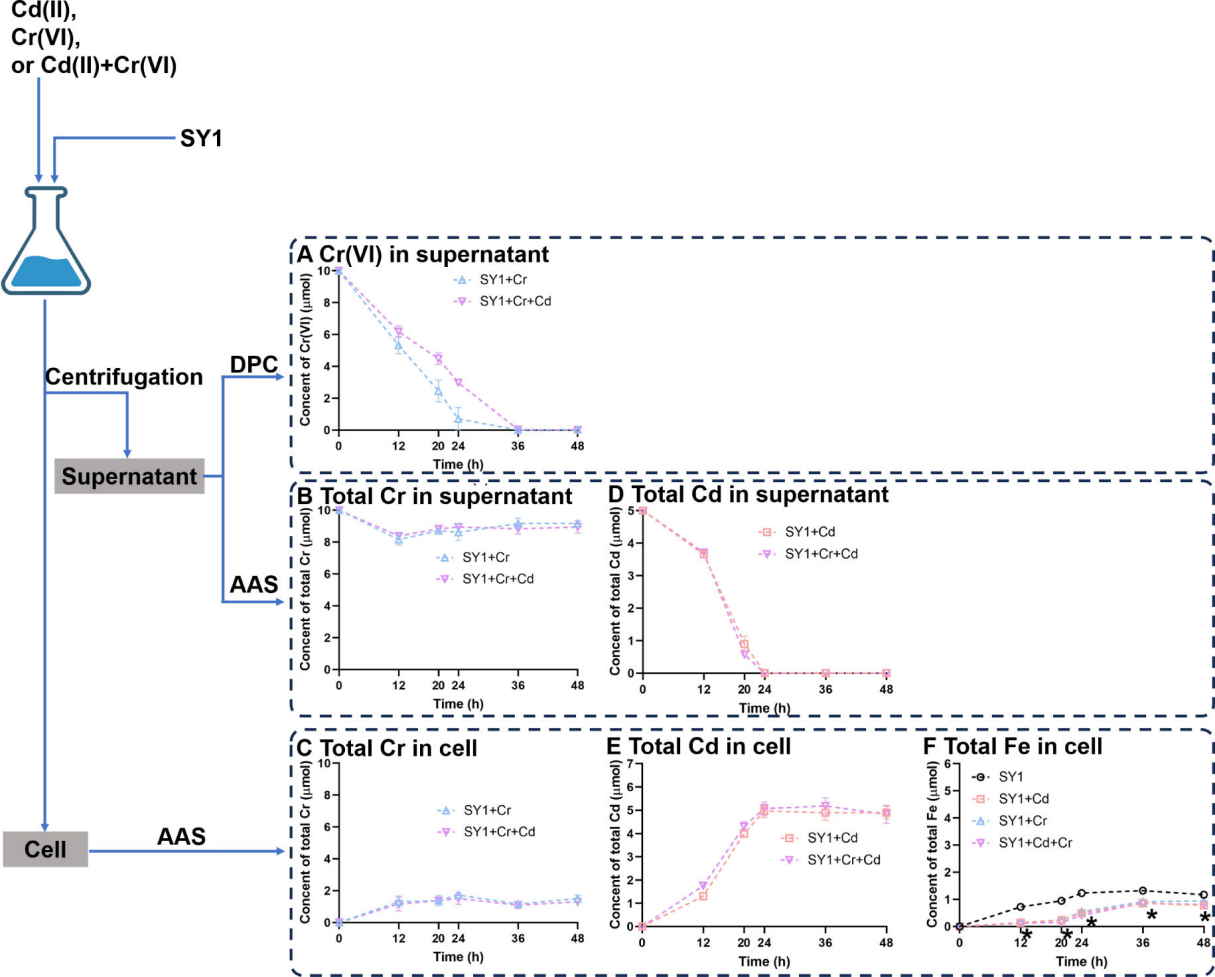
Cd(II) adsorption and Cr(VI) reduction of *Stenotrophomonas* sp. SY1. The strain SY1 was cultivated in LB medium with no heavy metal, Cd(II), Cr(VI), or Cd(II) + Cr(VI). (**A**) The changes in Cr(VI) content in the supernatant were measured using DPC. (**B**) The changes in total Cr content in the supernatant were measured using AAS. (**C**) The changes in total Cr content within the cells were measured using AAS. (**D**) The changes in total Cd content in the supernatant were measured using AAS. (**E**) The changes in total Cd within the cells were measured using AAS. (**F**) The changes in total Fe content within the cells were measured using AAS. * Indicates significance differences between experimental groups SY1 + Cd, SY1 + Cr, or SY1 + Cd + Cr compared to control group SY1 at each sampling time point (*P* < 0.05). These data represent average values obtained from three biological replicates.

The ability of *Stenotrophomonas* sp. SY1 to adsorb Cd(II) and reduce Cr(VI) was assessed in this study. The total content of Cd or Cr was determined using AAS, while DPC was used to measure the content of Cr(VI) in the supernatant. Our results showed that within 36 h, DPC analysis indicated complete deceases of Cr(VI) in the supernatant (Fig. 1A). However, according to AAS measurements, strain SY1 took up 10.95%–17.26% of the total Cr (Fig. 1C), while the remaining 81.67%–91.67% of it was found in the supernatant (Fig. 1B). On the other hand, AAS analysis revealed that a significant portion of total Cd present initially in the supernatant had been taken up by cells within 24 h (Fig. 1D and E).

During the entire cultivation of strain SY1, the cellular Fe content when exposed to Cd(II), Cr(VI), or Cd(II) + Cr(VI) was found to be lower compared to the control group (strain SY1 without heavy metals; Fig. 1F). At an early stage (12 h), the experimental group exhibited only about 20% of the Fe content observed in the control group (Fig. 1F). Subsequently, there was a gradual increase in the Fe content of the experimental group, reaching approximately 70% of that observed in the control group (Fig. 1F). The AAS data have been provided as supplementary material in Table S1.

### Exposure of SY1 to Cd(II) + Cr(VI) affects Fe-related metabolism

The aforementioned results demonstrated that strain SY1 exhibited the capability to adsorb Cd(II) and simultaneously reduce Cr(VI) (Fig. 1). Therefore, this study aimed to investigate the underlying mechanism in strain SY1 responsible for facilitating both Cd(II) adsorption and Cr(VI) reduction. We further investigated the responses of strain SY1 to Cd(II) + Cr(VI) exposure using proteomics approaches. Due to the high-efficiency of strain SY1 in Cd(II) adsorption and Cr(VI) reduction at 20 h (Fig. 1), samples for proteomic approaches were collected at this specific time point. Compared to the control group without additional heavy metals, treatment groups exposed to Cd(II) + Cr(VI) showed an increase in the relative abundance of 78 proteins and a decrease in the relative abundance of 142 proteins. The altered protein abundances were primarily enriched in biological processes such as secondary metabolite biosynthesis, cofactor biosynthesis, carbon metabolism, tricarboxylic acid (TCA) cycle, ABC transporters, and oxidative phosphorylation (Fig. S5).

**Fig 2:**
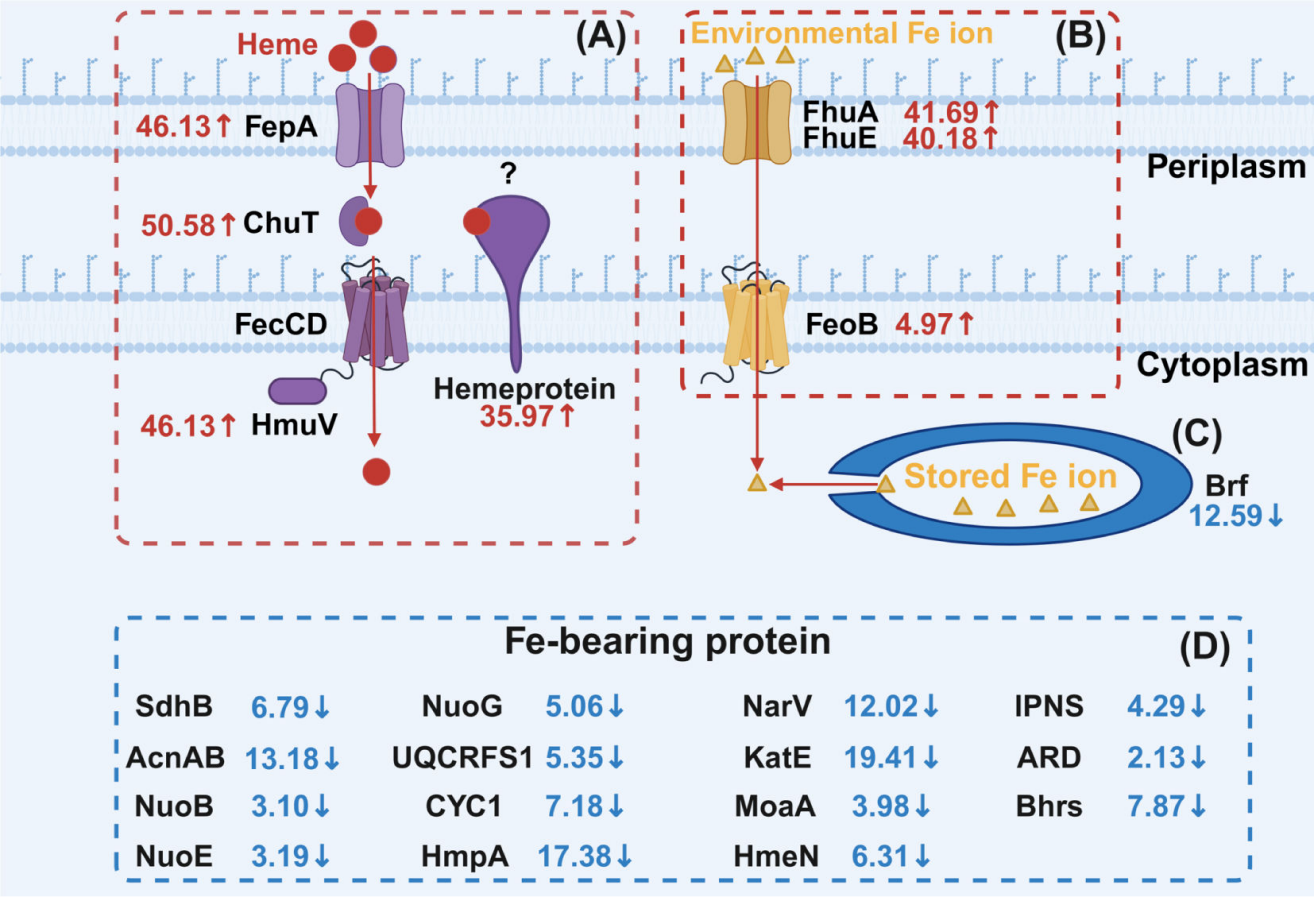
The abundance changes of Fe-related protein in *Stenotrophomonas* sp. SY1 on exposure to Cd(II) + Cr(VI). The experimental group consisted of strain SY1 cultivated in LB medium supplemented with Cd(II) +Cr (VI), while the control group comprised strain SY1 cultivated in LB medium without any heavy metal. (**A**) Four heme-uptake transporters and a hemeprotein. (**B**) Fe ion-uptake transporters. (**C**) Fe storage protein. (**D**) Fe-bearing protein.

Among them, the Fe-uptake transporters, including heme-uptake (FepA, ChuT, and HmuV) and Fe ion-uptake transporters (FhuA and FhuE), exhibited an increase in expression levels (Fig. 2B; Table S2). This upregulation facilitated bacteria to acquire a greater amount of iron from the surrounding environment (19, 50). Conversely, the Fe storage protein bacterioferritin (Bfr) showed a decrease in expression levels (Fig. 2C; Table S2), indicating that strain SY1 was experiencing iron deficiency (51). Several proteins containing iron as a cofactor or prosthetic group were downregulated (Fig. 2D; Table S2). These proteins play crucial roles with iron at their active sites. Specifically, NuoG, NuoE, NuoB, UQCRFS1, and CYC1 are involved in oxidative phosphorylation; CYC1 utilizes heme as its active site, while NuoG, NuoE, NuoB, and UQCRFS1 have Fe-S clusters as their active sites (52–54). The AcnA, AcnB, and SdhB proteins, which contain Fe-S clusters, contribute to the TCA cycle (55, 56). The MoaA protein and HemN protein play roles in the synthesis of molybdenum cofactors and heme metabolism, respectively (57, 58). The HmpA protein, NarV protein, and KatE protein utilize heme as their active sites to participate in NO detoxification, nitrate reduction, and hydrogen peroxide decomposition processes, respectively (59–61). IPNS is an abbreviation for isopenicillin N synthase. In the enzyme’s active site formation process, the Fe atom binds with its amino acid residues (62). Acireductone dioxygenase (ARD) plays a part in methionine synthesis. Its chemistry is determined by the identity of the Fe(II) ion present in its active site (63). Bhrs are a type of di-Fe proteins that contribute to oxygen-sensing capabilities as well as metal binding and antibiotic-resistance properties (64). During Fe-starvation of bacteria, less important Fe-bearing proteins are downregulated to reduce bacterial Fe consumption (19, 50). However, a specific Fe-bearing hemeprotein known as HhuH exhibited a significant increase, indicating that strain SY1 prioritized the heme protein HhuH in response to exposure to Cd(II) and Cr(VI) (Fig. 2; Table S2).

The molecular weight of the 460 aa HhuH protein was determined to be 48.06 KDa. According to TMHMM-2.0 (65), HhuH is predicted to be an inner membrane protein, with residues 1–12, 13–32, and 33–460 annotated as inside, TMhelix, and outside regions, respectively (Fig. S6). SignalP-6.0 (66) predicted that the signal peptide of HhuH belongs to the Sec/SPI type with a likelihood score of 0.9885 (Fig. S6). To confirm the localization of HhuH protein, different cellular components were extracted as shown in Fig. S7A. The presence of HhuH in these components was validated using ELISA (Fig. S7). The color development results from ELISA indicated that HhuH protein is present in both cytoplasm and cytomembrane compartments (Fig. S7B). Relative quantitative analysis by ELISA revealed that the abundance of HhuH in the cytoplasm was higher than its abundance in periplasm by 262.64% (Fig. S7C), suggesting that the main accumulation site for HhuH protein is within the cytoplasm.

Additionally, the pfam database (67) annotates that the 176–453 aa domain of HhuH is predicted to be the C-terminal (heme d1) domain of cytochrome cd1, a hemoprotein known as a nitrite reductase. Previous studies (68, 69) have demonstrated that cytochrome cd1 possesses two distinct heme domains: one heme c domain and one heme d1 domain. The function of nitrite reduction is performed by the heme d1 domain of cytochrome cd1 (68, 69). Therefore, it can be inferred that HhuH, which contains an annotated heme d1 domain, may also possess nitrite reductase activity similar to cytochrome cd1.

### The heme-uptake gene cluster is critical for adsorbing Cd(II) and reducing Cr(VI)

Based on the aforementioned results, Cd(II) and Cr(VI) activated the expression of Fe-uptake transporters, including heme-uptake transporters and Fe ion-uptake transporters (Fig. 2; Table S2). Among these, the genes encoding heme-uptake transporters are densely arranged in a specific gene cluster (*huu*), comprising four heme-uptake transporter genes and a hemeprotein gene (Fig. S8). The *huu* cluster is widely distributed and has been found in bacteria belonging to *Stenotrophomonas*, *Pseudomonas*, *Pantoea*, *Luteimonas*, and *Pseudoxanthomonas* genera (Fig. S8). The hemeprotein gene *hhuH* shows high conservation within the *huu* cluster across different bacterial species (Fig. S8).

**Fig 3:**
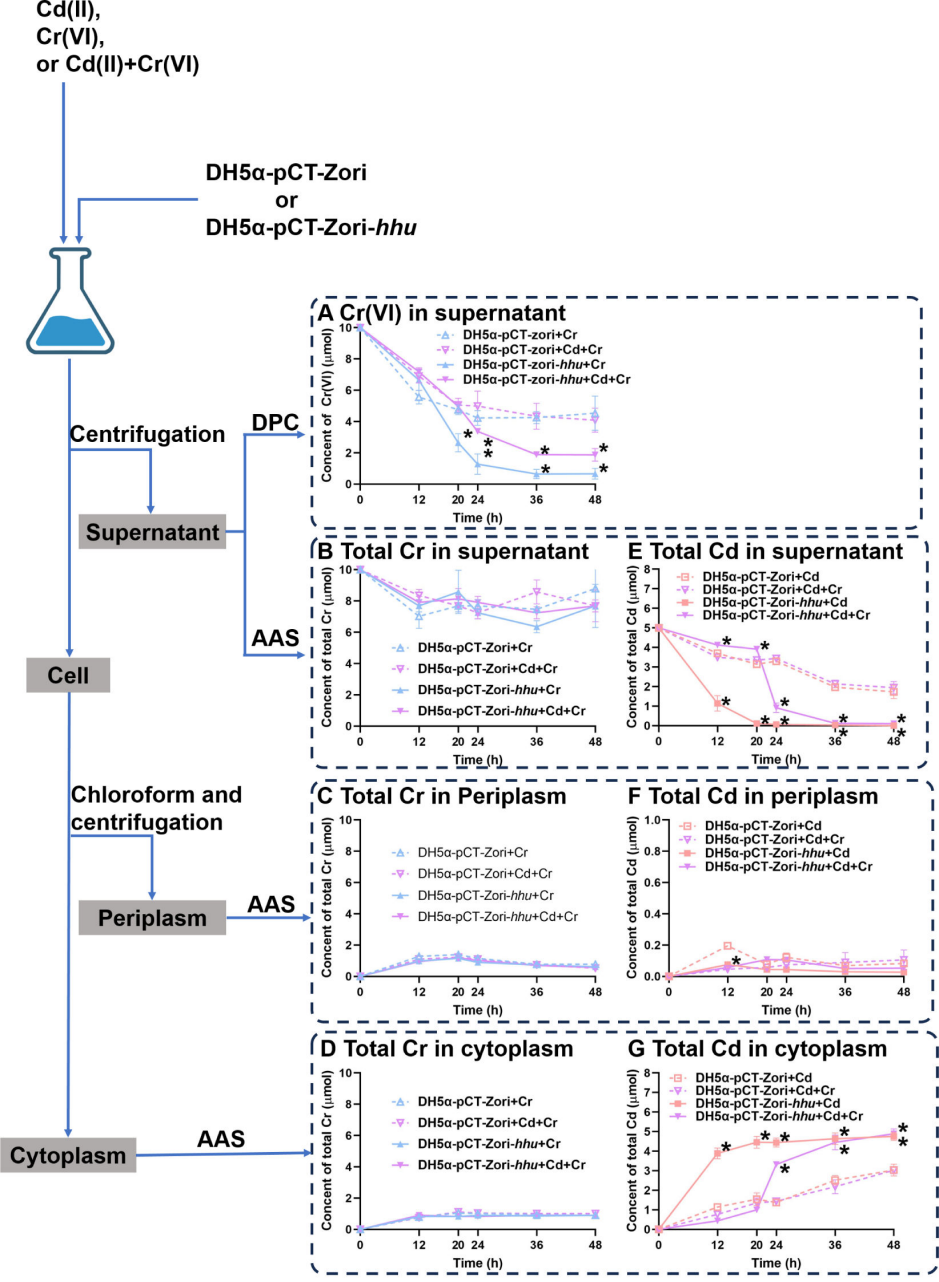
Roles of the heme-uptake gene cluster *hhu* in Cd(II) adsorption and Cr(VI) reduction. The DH5α-pCT-Zori-hhu strain (experimental group) or DH5α-pCT-Zori strain (control group) were cultured in LB medium supplemented with Cd(II), Cr(VI), or a combination of Cd(II) and Cr(VI). (**A**) The changes in Cr(VI) content in the supernatant were measured using DPC. (**B**) The changes in total Cr content in the supernatant were measured using AAS. (**C**) The changes in total Cr content in the periplasm were measured using AAS. (**D**) The changes in total Cr content in the cytoplasm were measured using AAS. (**E**) The changes in total Cd content in the supernatant were measured using AAS. (**F**) The changes in total Fe content in the periplasm were measured using AAS. (**G**) The changes of total Fe content in the cytoplasm were measured using AAS. These data represent average values obtained from three biological replicates. * Indicates significance differences between experimental groups DH5α-pCT-Zori-*hhu* compared to control group DH5α-pCT-Zori at each sampling time point (*P* < 0.05).

The *hhu* cluster was heterologously expressed in the *E. coli* DH5α strain, which lacks a similar gene cluster, to investigate its function. Compared with the transgene-free DH5α strain, the expression of the *hhu* cluster resulted in a significant increase in its ability to decrease Cd(II) and Cr(VI) in the supernatant by 41.88%–67.73% and 37.38%–70.98%, respectively, during the 48 h experiment (Fig. 3A and E). The concentration of Cr(VI) gradually decreased in the supernatant, while total Cr remained present (Fig. 3A through D). The heterologous expression of the *hhu* cluster enhanced Cr(VI) reduction (Fig. 3A and B). The majority of disappeared Cd(II) from the supernatant accumulated in cytoplasm, with only a small amount adsorbed in periplasm (Fig. 3E through G). The *hhu* cluster strengthened Cd(II) accumulation within cytoplasm but had little influence on Cd(II) accumulation within periplasm (Fig. 3F and G).

To investigate the involvement of Fe ion-uptake transporters in Cd(II) adsorption and Cr(VI) reduction, LB medium was supplemented with 50 µM FeCl_3_. The addition of 50 µM FeCl_3_ to the LB medium significantly enhanced the Cd(II) removal ability of strain SY1 in the supernatant, with an average increase of 7.73%–15.17% during the entire 48 h experiment (Fig. 1A; Fig. S8A). According to DPC analysis, strain SY1 supplemented with 50 µM FeCl3 exhibited an average efficiency improvement of 18.20%–33.93% in reducing Cr(VI) in the supernatant throughout the entire 48 h experiment (Fig. 1C; Fig. S8C). Furthermore, a substantial portion of total Cd was absorbed by strain SY1 from the supernatant (Fig. S9A and D), indicating that adsorption remained as the primary mechanism for Cd(II) removal by strain SY1 even in the presence of Fe(III). Although Cr(VI) concentration reached zero in the supernatant (Fig. S9C), most of the total Cr still remained in solution (Fig. S9B and E), suggesting that strain SY1 predominantly employed Cr(VI) reduction rather than absorption when supplemented with Fe(III). However, the Fe content of strain SY1 was increased by supplementing the medium with Fe(III) (Fig. 1F; Fig. S8F). The addition of Fe(III) alleviated the Fe starvation phenotype in strain SY1 exposed to heavy metals (Fig. 1F; Fig. S8F).

When strain SY1 was cultivated in LB under different conditions, the transcriptional changes of gene *hhuH* were measured using RT-qPCR. The results showed that the transcription of gene *hhuH* in strain SY1 was positively correlated with the presence of Cr(VI) in the medium (Fig. S10). Compared to the control group (strain SY1 without any metal exposure), both Cr(VI) and Cd(II) + Cr(VI) induced an up-regulation of gene *hhuH* expression during the early phase (Fig. S10A and B). As the concentration of Cr(VI) in the supernatant decreased (Fig. 1A; Fig. S8A), there was a corresponding downregulation of its transcriptional activity (Fig. S10C through E). Furthermore, it was observed that the addition of Fe(III) resulted in lower levels of gene *hhuH* expression compared to samples without Fe(III) supplementation (Fig. S10). Additionally, MIC experiments demonstrated that Fe(III) supplementation enhanced strain SY1’s tolerance toward Cd(II) or Cr(VI) toxicity (Fig. S3).

### The hemeprotein HhuH chelates Cd(II) and reduces Cr(VI)

**Fig 4:**
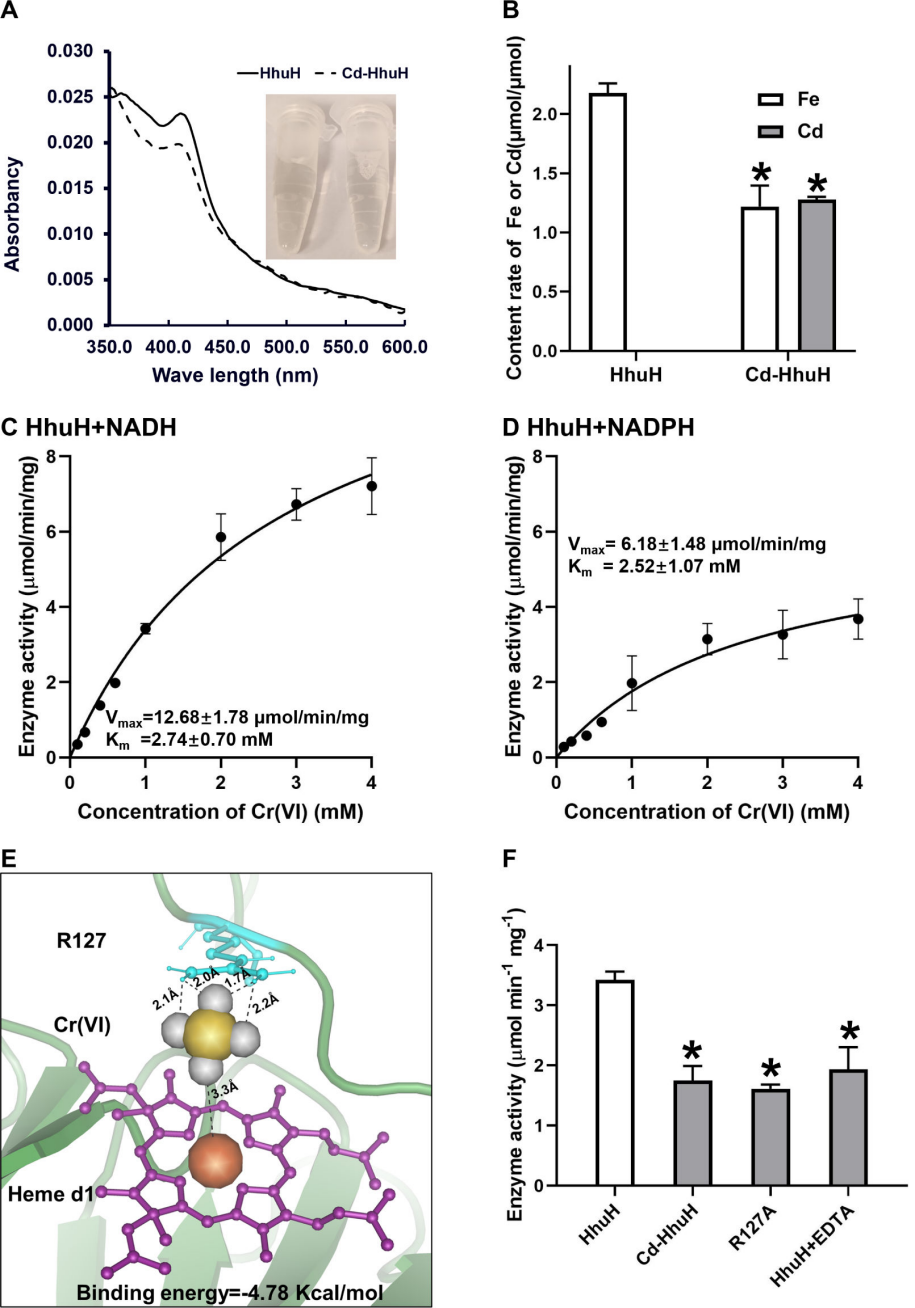
Characterization and enzyme activity of HhuH. (**A**) The absorption spectrum of the purified HhuH protein was measured after incubation with or without Cd(II). (**B**) The Fe or Cd content rate of the purified HhuH protein was quantified following incubation with or without Cd(II). The data represent the average values obtained from three biological replicates, and * indicates a significant difference in Cd-HhuH compared to HhuH in terms of Fe or Cd content rate (*P* < 0.05). The enzyme activity curve of HhuH catalyzes the reduction of Cr(VI) using either (**C**) NADH or (**D**) NADPH. The data represent the average values obtained from three biological replicates. (**E**) Molecular docking of Cr(VI) with HhuH. Purple: porphyrin ring of heme d1; orange: Fe(II) atom of heme d1; yellow: Cr atom of CrO_4_^2−^; gray: O atom of CrO_4_^2−^; blue: R127 of HhuH. (**F**) The Cr(VI) reduction activities of HhuH, Cd-binding protein Cd-HhuH, mutant protein R127A, and EDTA-inhibited HhuH. The data represent the average values obtained from three biological replicates, and * indicates a significant difference in the Cr(VI) reduction activities of experimental group Cd-HhuH, R127A, or HhuH + EDTA compared to control group HhuH (*P* < 0.05).

The phylogenetic analysis of HhuH with other hemeproteins revealed that it is genetically distinct from known hemeproteins, with cytochrome *cd1* being its closest relative (Fig. S11A). The prediction of conservative motifs indicated that HhuH possesses some different motifs compared with cytochrome *cd1* (Fig. S11B). The HhuH protein was purified using a Ni column in this study. Subsequently, the SDS-PAGE gel of the purified product revealed a single protein band (with a size range between 43 and 55 KDa) that was confirmed to be hemeprotein HhuH with His-tag (51.24 KDa; Fig. S12). Furthermore, LC-MS analysis of the purified product indicated that the relative amount of HhuH was 90.28%, and relative amounts of single impurity were less than 2.18% (Fig. S12). Therefore, this purified product can be identified as the HhuH protein. The HhuH protein exhibited a faint yellow color with an absorption peak at ~410 nm (Fig. 4A). These characteristics were consistent with heme *d1*, a type of Fe(II)-porphyrin commonly utilized as the cofactor for cytochrome *cd1* (69, 70). Therefore, it is likely that heme *d1* serves as the cofactor for the HhuH hemeprotein. Additionally, the presence of heme in hemeproteins can be authenticated using the ABTS chromogenic method (70). The positive phenotype displayed by HhuH in the ABTS measurement, similar to that of standard hemoglobin (the positive control protein; Fig. S13), confirms the existence of heme in the HhuH protein. Furthermore, based on its amino acid sequence, it is predicted that the HhuH protein possesses the heme d1 domain, which has been reported to mediate nitrite reduction in other hemeproteins (68, 69). The purified HhuH demonstrated its ability to reduce nitrite (Fig. S14), thereby confirming its possession of this heme d1 domain.

The function of HhuH was investigated by conducting interaction experiments with Cd(II). The results revealed that the addition of Cd(II) reduced color intensity and flattened the absorption peak of HhuH (Fig. 4A). The analysis of Cd and Fe content revealed that one unit of HhuH contains approximately two units of Fe, whereas one unit of Cd-HhuH contains one unit each of Fe and Cd (Fig. 4B), indicating that one HhuH unit may possess two units of heme *d1*, where the interaction between Cd and heme *d1* leads to the replacement of Fe(II) by Cd(II) in one unit. Previous studies have also shown this heme-mediated substitution phenomenon in other hemeproteins, confirming that Cd(II) is chelated with porphyrin from heme after this replacement (71, 72). Thus, it can be inferred that HhuH detoxified Cd(II) via heme-mediated chelation.

The Cr(VI)-reduction experiments of purified HhuH were conducted, revealing that HhuH employs NADH or NADPH as electron donors for the reduction of Cr(VI), thereby demonstrating its capacity to detoxify Cr(VI) through reduction (Fig. 4C and D). In the absence of the enzyme, there was minimal direct reduction of Cr(VI) by NADH or NADPH (Fig. S15). When in a free electron donor state, HhuH exhibited limited ability to reduce Cr(VI) (Fig. S15). Compared with NADPH, using NADH resulted in higher enzyme activity for HhuH, while there was little difference in the K_m_ value between them (Fig. 4C and D). Thus, NADH is a more suitable electron donor for HhuH in reducing Cr(VI) than NADPH. Additionally, HhuH exhibited a lower K_m_ value in nitrite reduction compared to Cr(VI) reduction (Fig. 4C and D; Fig. S12), indicating that nitrite is the preferred substrate for the HhuH protein over Cr(VI). Phylogenetic analysis of HhuH with other Cr(VI)-reductases revealed its independent evolutionary lineage from known Cr(VI)-reductases, with the closest genetic relationship observed between HhuH and the Cr(VI)-reductase OmcA/MtrC (Fig. S16A). Furthermore, a comparison of conservative motifs indicated distinct differences between HhuH and the Cr(VI)-reductase OmcA/MtrC regarding their conserved sequence patterns (Fig. S16B). Additionally, differences were observed in the characterization of Cr(VI)-reduction-related properties between HhuH and other reductases (Table 2), highlighting its novelty as a Cr(VI)-reductase.

**TABLE 2:** Cr(VI)-reduction-related characterization of hemeprotein HhuH and other Cr(VI)-reductases

Reductase	Cofactors	K_m_ (μmol/L)	V_max_ (μmol/min/mg)	Reference
ChrR	FMN	260	8.80	(73)
YieF	FMN	200	5.00	(73)
NemA	FMN	212	0.95	(44)
NfoR	FMN	422	52.08	(74)
OYE family	FMN	8	16.00	(75, 76)
NfsA	FMN	36	0.25	(77)
CsrF	FMN	250.6	11.92	(78)
FesR	FAD and Fe-S cluster	1,682	4.06	(79)
MtrC	Heme c	34	1.01	(80)
OmcA	Heme c	41	7.78	(80)
**HhuH**	**Heme d1**	**2,740**	**12.68**	**This study**

### Role of R127 and Fe(II) in Cr(VI) reduction by HhuH

To explore the mechanism of Cr(VI) reduction in HhuH, molecular docking was performed to predict the interaction site between Cr(VI) and HhuH (46). This prediction revealed that HhuH bound Cr(VI) with a binding energy of −4.78 kcal/mol (Fig. 4E). Cr(VI) typically existed as the oxyanion form of CrO_4_^2–^, composed of one Cr atom and four O atoms (81). Among these O atoms, three were involved in hydrogen bonding with two amino groups of R127, with bond lengths ranging from 1.7 to 2.2 Å (Fig. 4E). Another O atom of Cr(VI) formed a linkage with Fe(II) in heme *d1* at a distance of 3.3 Å (Fig. 4E). Thus, R127 and Fe(II) were identified as crucial interaction sites between HhuH and Cr(VI).

To further explore the role of R127, we generated a site-directed mutant, replacing R127 with alanine (resulting in R127A) in HhuH. The 3D structure of the mutant R127A was compared to that of the wild-type HhuH using PyMOL software. The resulting analysis revealed an RMSD value of 0.038 Å, indicating a negligible deviation between these two protein structures (47). Therefore, it can be concluded that the substitution of R127 has minimal impact on the overall 3D structure of HhuH. Additionally, we replaced Fe(II) with Cd(II) through the interaction between HhuH and Cd(II) (Cd-HhuH). Compared to the wild-type HhuH, both the mutant protein R127A and Cd-HhuH exhibited a decrease in Cr(VI)-reduction enzyme activity by 48.83% and 52.92%, respectively (Fig. 4F). These findings support the critical role of the R127 and Fe(II) in Cr(VI) reduction by HhuH. Furthermore, it is observed that the processes of Cd(II)-binding and Cr(VI)-reduction mediated by HhuH compete with each other due to their reliance on the same cofactor heme *d1*. This can account for the findings depicted in Fig. 3A and G, which demonstrate that the strain DH5α-pCT-zori-*hhu* exhibited diminished Cd(II) uptake or Cr(VI) reduction in the presence of both Cd(II) and Cr(VI), compared to its phenotype under individual Cd(II) or Cr(VI) conditions. Additionally, the use of EDTA as an inhibitor resulted in a 43.42% reduction in the enzymatic activity of HhuH involved in Cr(VI) reduction. EDTA is a metal-chelating agent that has been previously reported to disrupt Cr(VI) reduction in other Cr(VI) reductases (81).

## DISCUSSION

Fe plays a pivotal role in the biogeochemical cycle of toxic heavy metals by reducing their mobility and toxicity (12, 75, 82). The dissimilatory capacity of Fe(III)-reducing bacteria enables the conversion of Fe(III) to Fe(II), indirectly facilitating Cr(VI) reduction via Fe(II) (75). Quinone-reducing bacteria utilize dissolved organic matter for Cr(VI) reduction, and the presence of Fe(III) enhances their electron transfer capability (82). Sulfate-reducing bacteria optimize the crystal structure of Fe minerals, thereby enhancing Cd(II)-binding sites for increased Cd(II) adsorption (12). In this study, we observed that Fe also facilitated Cd(II) adsorption and Cr(VI) reduction in *Stenotrophomonas* sp. SY1.

Conversely, under heavy metal exposure, the Fe content within bacteria is found to be limited (14, 18). During Fe starvation, bacteria prefer upregulated heme, highlighting the significant role of heme in the metabolism of Fe and heavy metals (15). Our experiment revealed that strain SY1 experienced Fe deficiency as a result of exposure to Cd(II) or Cr(VI). Subsequent experiments confirmed the preferential utilization of Fe from other Fe-bearing proteins by strain SY1 for synthesizing HhuH. This synthesized HhuH was then utilized for adsorbing Cd(II) and reducing Cr(VI). The toxicity of Cd(II) is closely associated with its concentration in the bio-available form, and intracellular accumulation (binding) represents one of the bacterial mechanisms for detoxifying Cd(II). The protein metallothionein, renowned for its ability to bind with Cd(II), enhances bacterial resistance against Cd(II) and facilitates the intracellular accumulation of Cd(II) within bacteria (83). In this study, *Stenotrophomonas* sp. SY1 possesses the hemeprotein HhuH, which exhibited a capacity to bind with Cd(II), enabling tolerance toward exposure to Cd(II).

**Fig 5:**
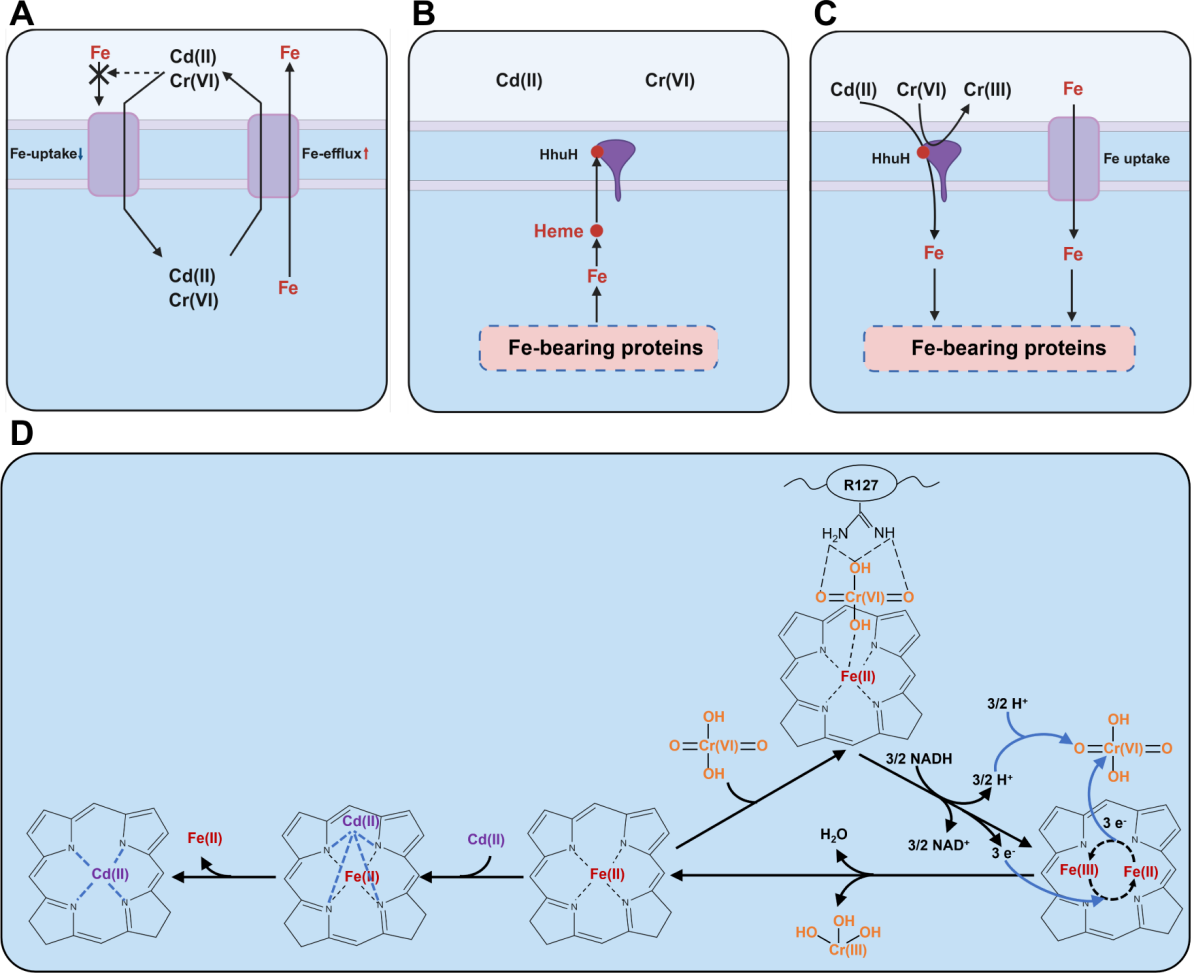
Proposed roles of the ferruginous hemeprotein HhuH in detoxifying Cd and Cr. (**A**) Fe starvation caused by Cd(II) and Cr(VI). (**B**) Bacterial behavior for heme preference. (**C**) Detoxication of the hemeprotein HhuH toward Cd(II) and Cr(VI). (**D**) The mechanism underlying heme-mediated Cd(II) chelation and Cr(VI) reduction.

Hemeprotein is a crucial and widely distributed Fe-bearing protein that participates in essential biological reactions, including electron transfer, drug detoxification, oxygen transport and storage, gene regulation, and signal transduction (84, 85). The electron transfer of hemeproteins involves the cofactor heme, which facilitates electron transfer by cycling the oxidation state of its Fe atom between Fe(II) and Fe(III) (82). In addition, certain hemeproteins are reportedly involved in Cr(VI) reduction (80, 86). For instance, the hemeproteins MtrC and OmcA can reduce Cr(VI) to Cr(III) through electron transfer (80). The hemeprotein NirS exhibits a strong correlation with Cr(VI) reduction during denitrification in *Pseudomonas* (86). In the enzymatic reduction of Cr(VI), NAD(P)H serves as the electron donor for certain Cr(VI) reductases (80). These NAD(P)H-dependent Cr(VI) reductases, including ChrR, YieF, and NfsA, function as two-electron reducers (80). Here, we discovered that the hemeprotein HhuH can reduce Cr(VI) to Cr(III). Further experiments verified the importance of R127 and heme *d1* as critical sites of HhuH for reducing Cr(VI). We propose that HhuH captures Cr(VI) through its R127 and heme *d1*. Subsequently, heme *d1* utilizes NADH for electron transfer to reduce Cr(VI) (Fig. 5D).

Heme is the essential cofactor of hemeprotein, formed by the chelation of Fe and porphyrin (87). Porphyrin coordinates with Fe and exhibits the ability to bind Cd (71, 72). Several studies have observed that Cd replaces Fe in heme, leading to the formation of Cd–porphyrin (71, 72). This substitution occurs because of the higher stability of Cd–porphyrin than Fe–porphyrin (88). Our study revealed that HhuH possesses heme *d1*. A series of experiments confirmed that HhuH can chelate Cd(II) through its heme *d1* moiety. We hypothesize that hemeprotein HhuH binds Cd(II) via its heme *d1* group and subsequently displaces Fe(II), ultimately resulting in the formation of a stable Cd–porphyrin (Fig. 5D).

## Data Availability

Data will be made available on request. The mass spectrometry proteomics data have been deposited to the ProteomeXchange Consortium (http://proteomecentral.proteomexchange.org) via the iProX partner repository with the identifier PXD050232.

## SUPPLEMENTAL MATERIAL

Supplemental material aem.02097-24-s0001.docx Table S2; Figures S1 to S16.

Table S1 aem.02097-24-s0002.xlsx Atomic absorption spectrum data.

ASM does not own the copyrights to Supplemental Material that may be linked to, or accessed through, an article. The authors have granted ASM a non-exclusive, world-wide license to publish the Supplemental Material files. Please contact the corresponding author directly for reuse.
