# Supplementary material for: Ferruginous hemeprotein HhuH facilitates the cadmium adsorption and chromium reduction in Stenotrophomonas sp. SY1
Source: Appl Environ Microbiol. 2024 Dec 4;91(1):e02097-24. doi: 10.1128/aem.02097-24 (PMC11784086; doi:10.1128/aem.02097-24)
Supplement: Supplemental material — Table S2; Figures S1 to S16. [file aem.02097-24-s0001.docx]

**Ferruginous hemeprotein HhuH** **facilitates the cadmium adsorption and chromium reduction in** ***Stenotrophomonas* sp. SY1**

Zijie Zhou^a^, Hongbo Yu^a^, Jiahui Liu^a^,Lin Zhu^a^, Gejiao Wang^a^, Kaixiang Shi^a^*

*a: National Key Laboratory of Agricultural Microbiology, College of Life Science and Technology, Huazhong Agricultural University, Wuhan 430070, China.*

** Corresponding authors:*

*Kaixiang Shi, kaixiangshi@mail.hzau.edu.cn, National Key Laboratory of Agricultural Microbiology, Huazhong Agricultural University, Wuhan 430070, China.*

**Table S2. Fe-related proteins of *Stenotrophomonas* sp. SY1 exposed to Cd(II) + Cr(VI).**

| **Number** | **Name** | **Description** | **change of relative abundance** |
| --- | --- | --- | --- |
| **Fe-uptake transporter** | | | |
| MCD9085853.1 | FepA | Outer membrane channel of heme uptake | +46.13 |
| MCD9085856.1 | ChuT | Heme binding periplasmic protein | +50.58 |
| MCD9085858.1 | Hmuv | Heme ABC transporter ATP-binding protein | +46.13 |
| MCD9087228.1 | FeoB | Ferrous iron transport protein | +4.97 |
| MCD9088497.1 | FhuA | TonB-dependent siderophore receptor | +41.69 |
| MCD9087859.1 | FhuA | TonB-dependent siderophore receptor | +10.47 |
| MCD9086461.1 | FhuE | Outer membrane siderophore receptor | +40.18 |
| **Fe storage protein** | | | |
| MCD9086331.1 | Bfr | Bacterioferritin, Fe storage | -11.59 |
| MCD9086416.1 | Bfr | Bacterioferritin, Fe storage | -4.70 |
| MCD9088353.1 | Bfr | Bacterioferritin, Fe storage | -7.87 |
| **Fe-bearing protein** | | | |
| MCD9085855.1 | HhuH | Hemeprotein | +35.97 |
| MCD9085452.1 | HmpA | NO-inducible flavohemoprotein | -11.38 |
| MCD9086976.1 | NarV | Respiratory nitrate reductase subunit gamma | -12.02 |
| MCD9087600.1 | KatE | Heme catalase | -19.41 |
| MCD9086496.1 | CYC1 | Cytochrome c1 | -7.80 |
| MCD9088745.1 | HemN | oxygen-independent coproporphyrinogen III oxidase | -6.31 |
| MCD9087206.1 | AcnA | Aconitate hydratase | -13.18 |
| MCD9087203.1 | AcnB | Bifunctional aconitate hydratase 2/2-methylisocitrate dehydratase | -2.03 |
| MCD9086803.1 | SdhB | Succinate dehydrogenase iron-sulfur subunit | -6.79 |
| MCD9086595.1 | NuoG | NADH-quinone oxidoreductase subunit | -5.06 |
| MCD9086593.1 | NuoE | NADH-quinone oxidoreductase subunit | -3.19 |
| MCD9086590.1 | NuoB | NADH-quinone oxidoreductase subunit | -3.10 |
| MCD9086494.1 | QCRFS1 | Ubiquinol-cytochrome c reductase iron-sulfur subunit | -5.35 |
| MCD9086965.1 | MoaA | GTP 3',8-cyclase | -3.98 |
| MCD9086586.1 | IPNS | Isopenicillin N synthase family oxygenase | -4.29 |
| MCD9087248.1 | ARD | Acireductone dioxygenase | -2.13 |
| MCD9086470.1 | Bhrs | Oxygen transportation | -7.87 |

**
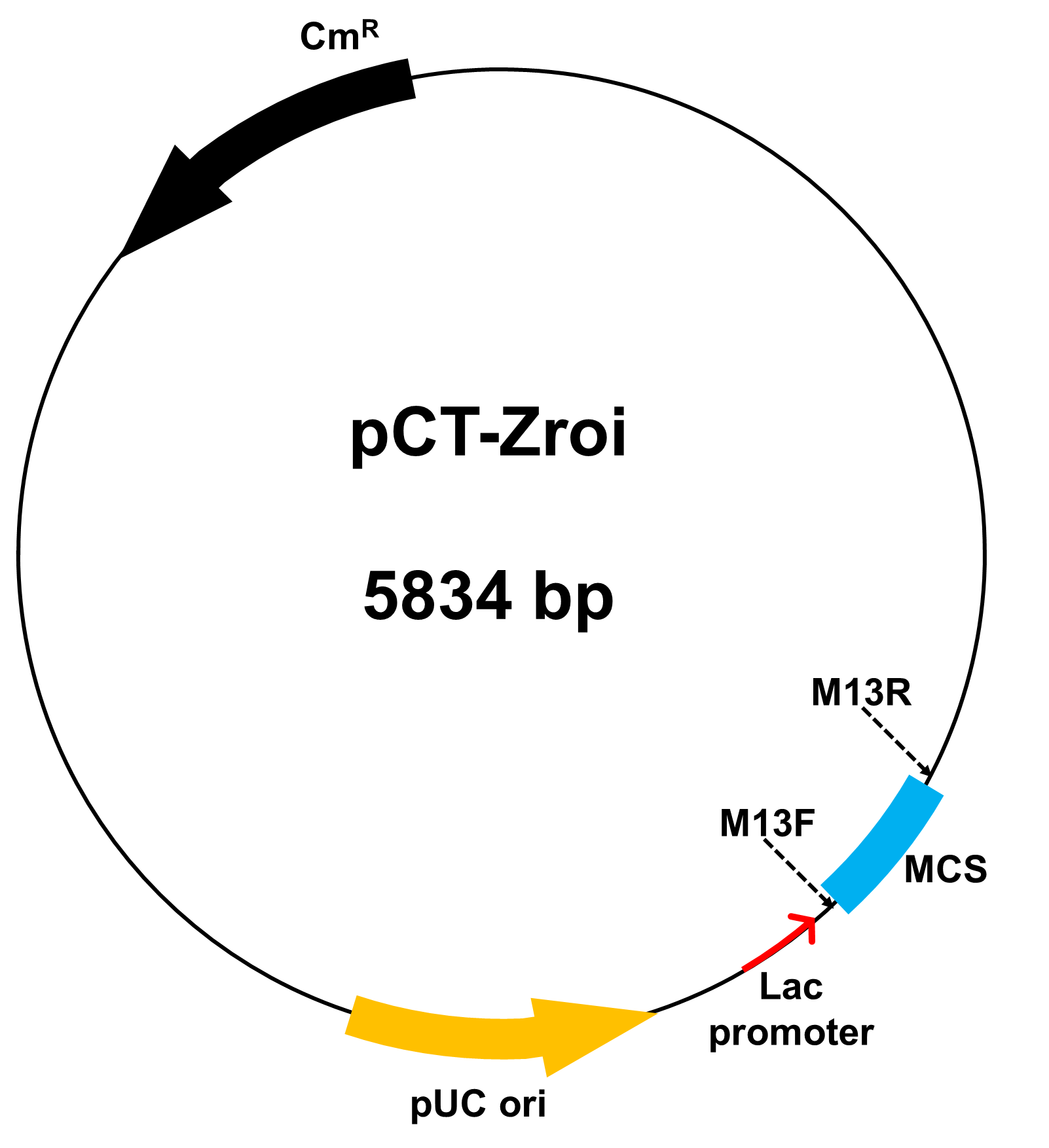
**

**Figure S1. The plasmid profile of heterologous expression vector pCT-Zori.** The multiple cloning site (MCS) serves as the insertion locus for target gene. The Lac promoter is employed for transcription initiation of the target gene. The pUC ori represents a high-copy-number replication origin specific to pUC plasmids. Cm^R^ demotes the chloramphenicol resistance gene utilized for antibiotic selection purposes. M13F/M13R are commonly used primer pairs for sequencing applications.


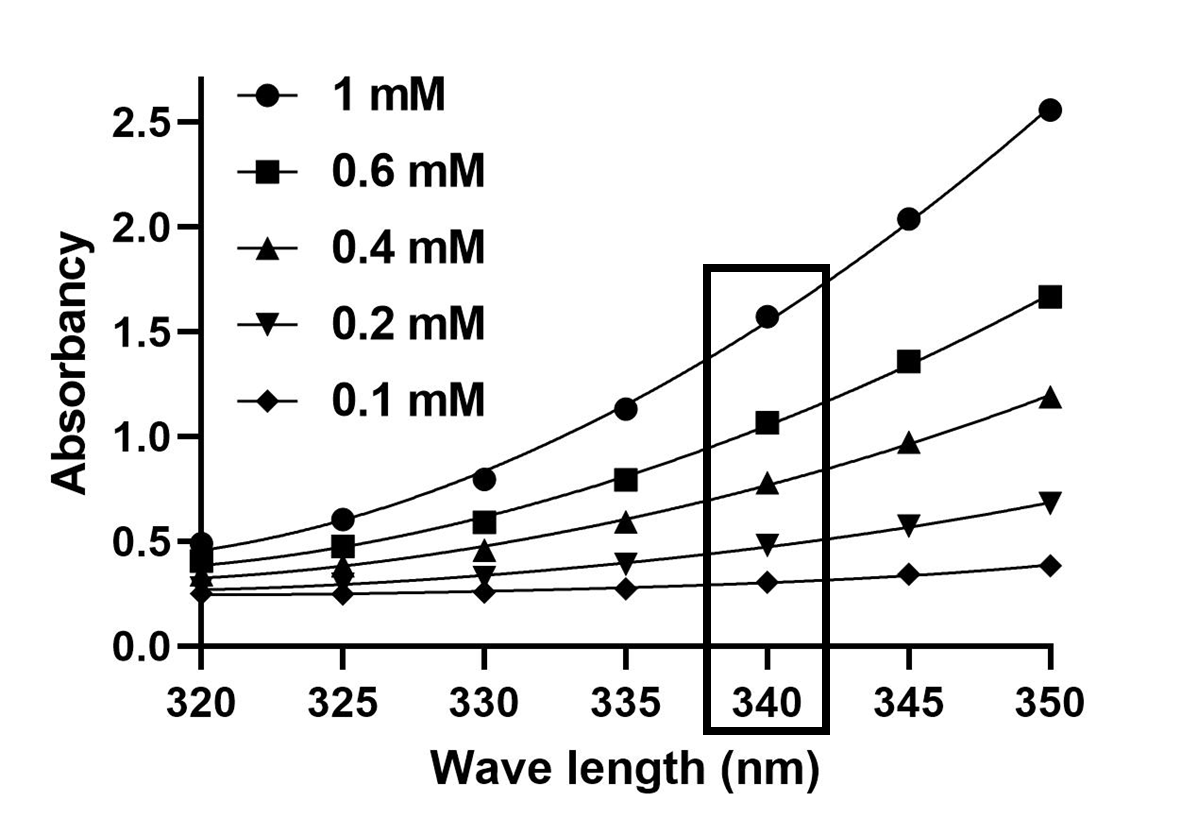


**Figure S2. The absorption spectrum of Cr(VI).** The absorption spectrum of Cr(VI) at different concentrations (0.1, 0.2, 0.4, 0.6, or 1 mM) was measured using the BioTek Cytation5 microplate reader (BioTek Instruments Co., Ltd., Vermont, USA). The calibration curve was drawn by GraphPad Prism software version 9.5.


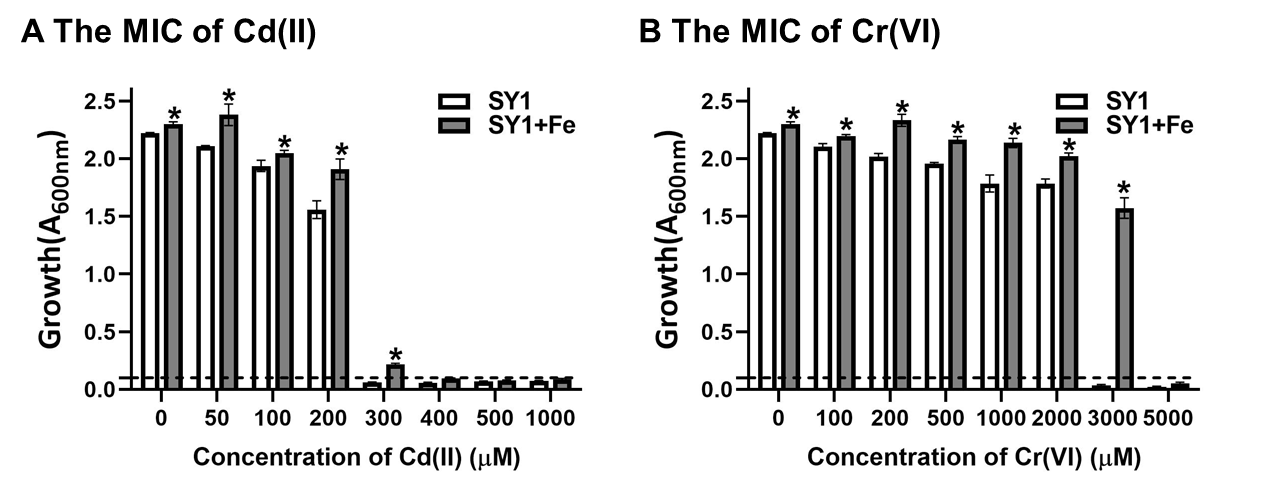


**Figure S3. The MIC of strain SY1 to Cd(II) or Cr(VI).** The dotted line, representing the OD_600_ value of 0.1, serves as the threshold for assessing growth and inhibition. The data presented here are the average values obtained from three biological replicates. The * indicating SY1+Fe group had a significantly different (p < 0.05) with SY1 group in OD_600_ value, when OD_600_ value of SY1+Fe group exceed 0.1.


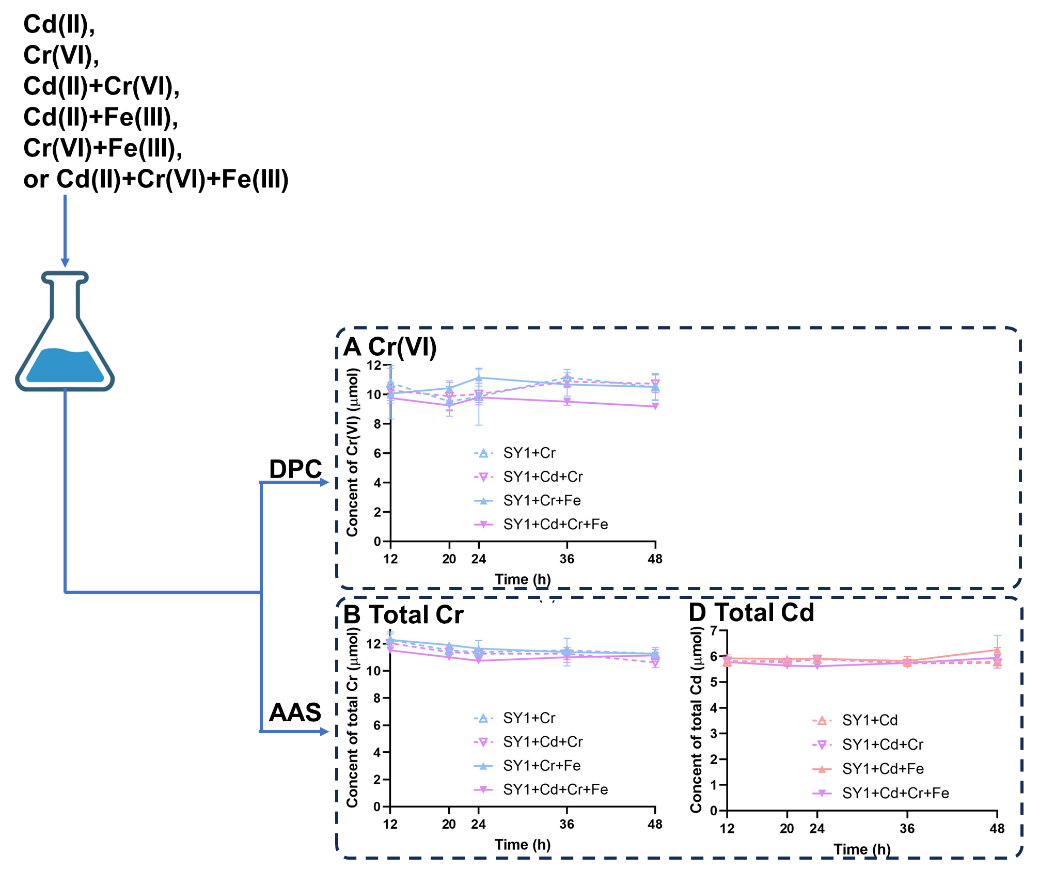


**Figure S4. The variation of heavy metal content in the control group without cells.** (A) The concentration of Cr(VI) in the no cell control group was measured using DPC. (B) The total Cr content was determined by AAS. (C) The total Cd content was analyzed using AAS.


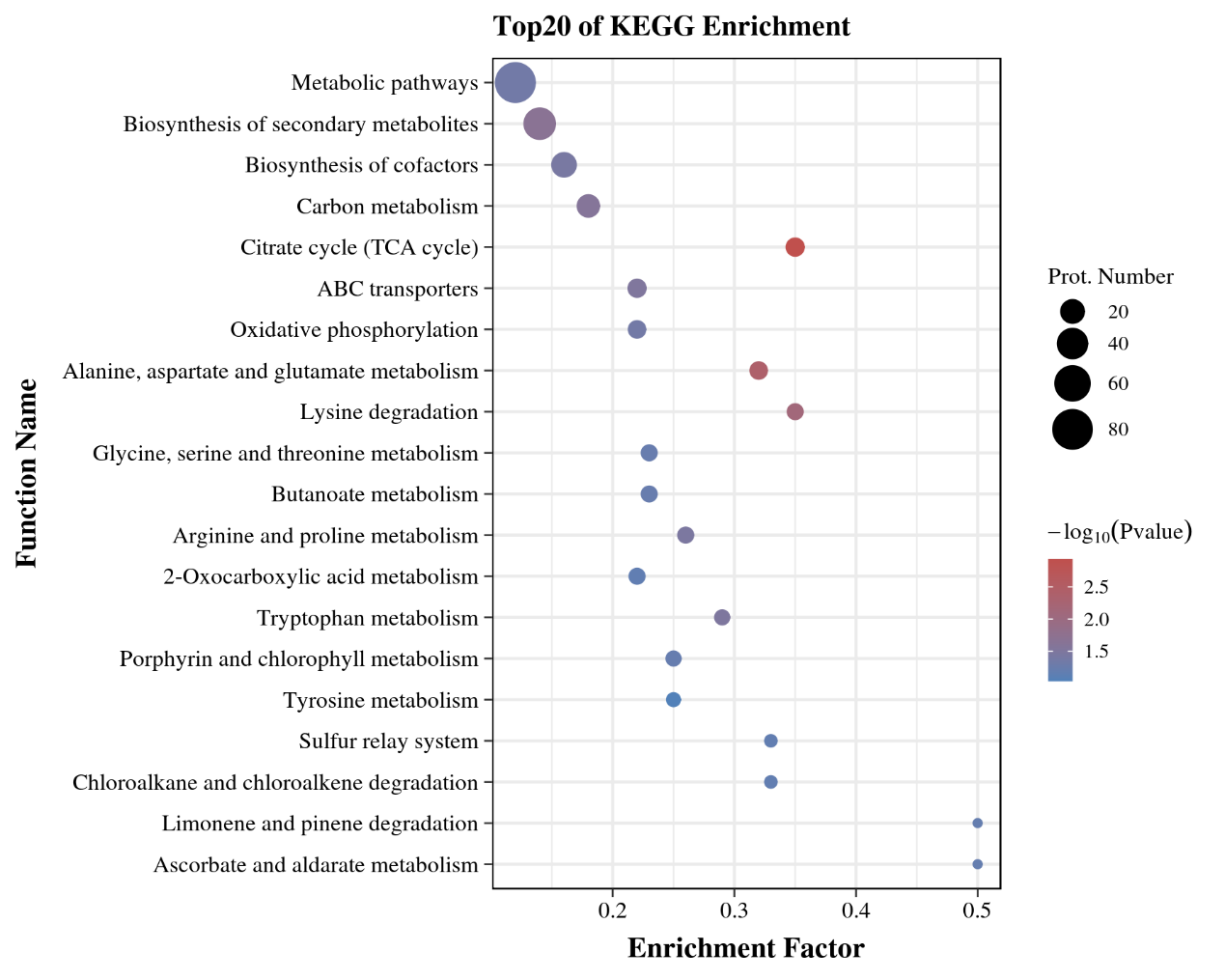


**Figure S5. KEGG pathways were compared with and without the addition of Cd(II) + Cr(VI) in *Stenotrophomonas* sp. SY1.**


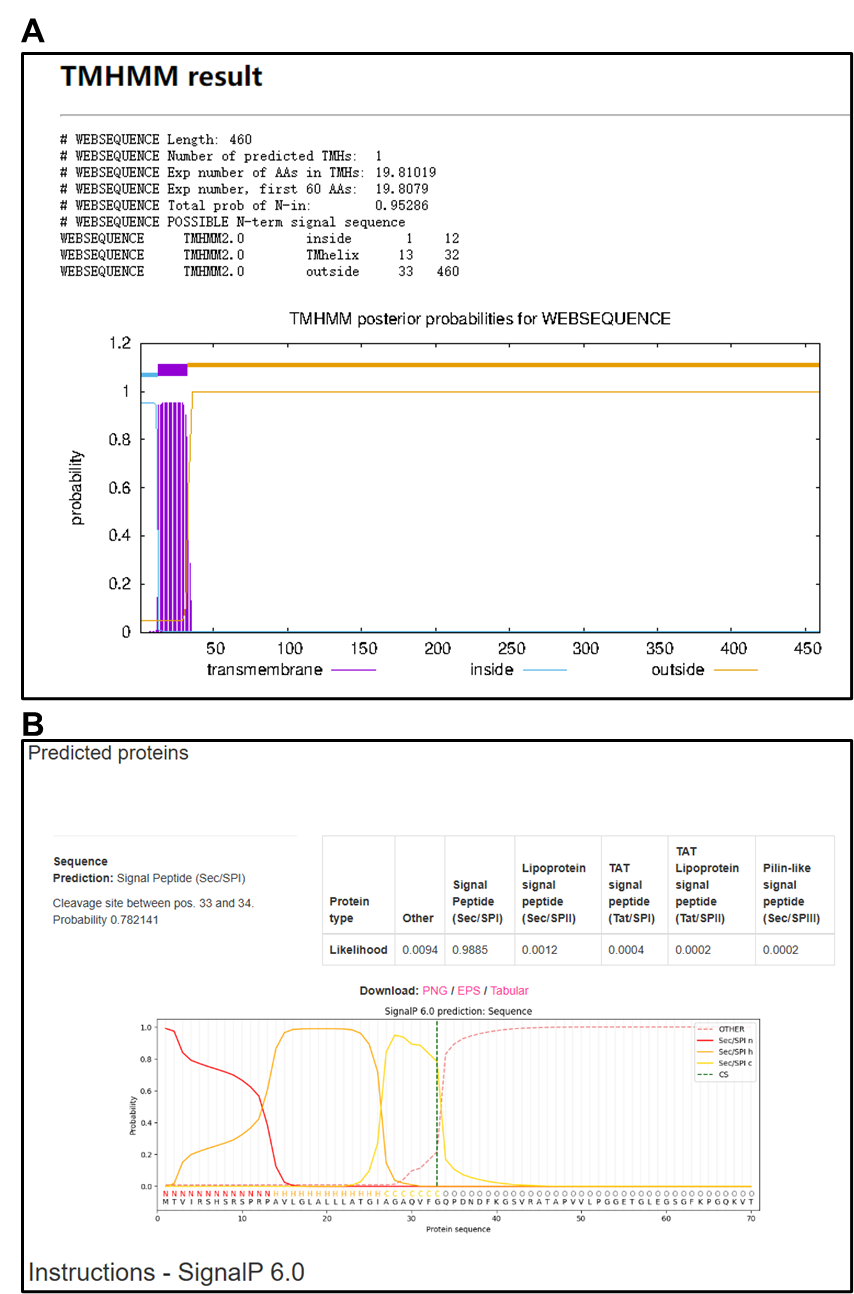


**Figure S6. The transmembrane and signal peptide prediction of hemeprotein HhuH. (A)** This transmembrane prediction was performed by TMHMM-2.0 (https://services.healthtech.dtu.dk/services/TMHMM-2.0/). **(B)** This signal peptide prediction was obtained from SignalP-6.0 (https://services.healthtech.dtu.dk/services/SignalP-6.0/).


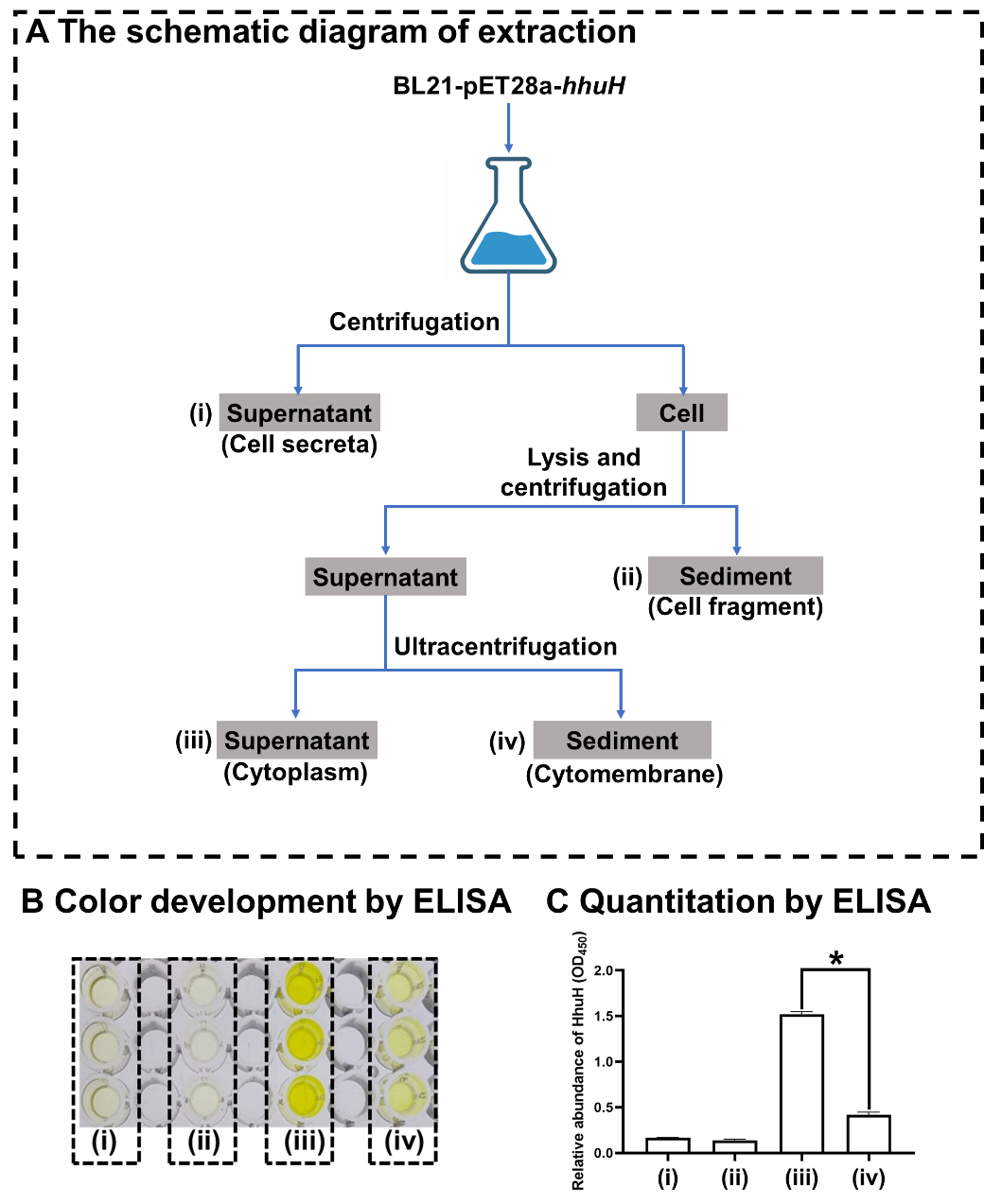


**Figure S7. The protein localization assays for HhuH. (A)** The schematic diagram illustrates the extraction process for different components, namely cell secretions **(i)**, cell fragments **(ii)**, cytoplasm **(iii)**, and cytomembranes **(iv)**. **(B)** The ELISA was used to measure the exist of hemeprotein HhuH in different components, with the development of yellow color indicating the existence of hemeprotein HhuH. **(C)** The relative quantitation of HhuH protein in these components was determined using ELISA assays. The data represent the average values obtained from three biological replicates, and * indicates a significance difference in the relative abundance of HhuH between the cytoplasm and cytomembrane (p < 0.05).


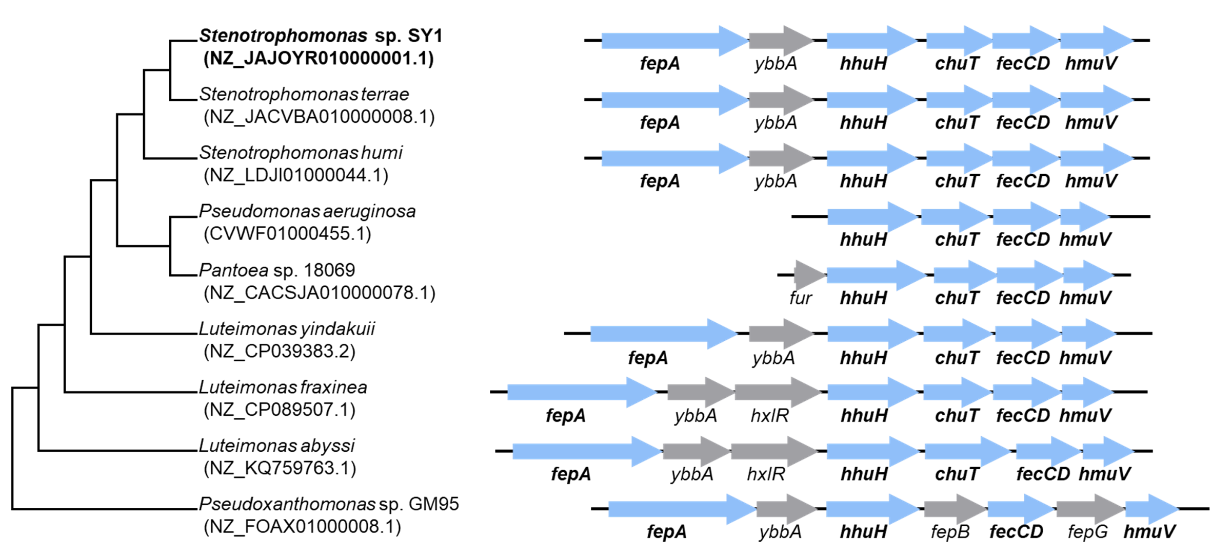


**Figure S8. The arrangement of the *hhu* gene cluster among different species.**


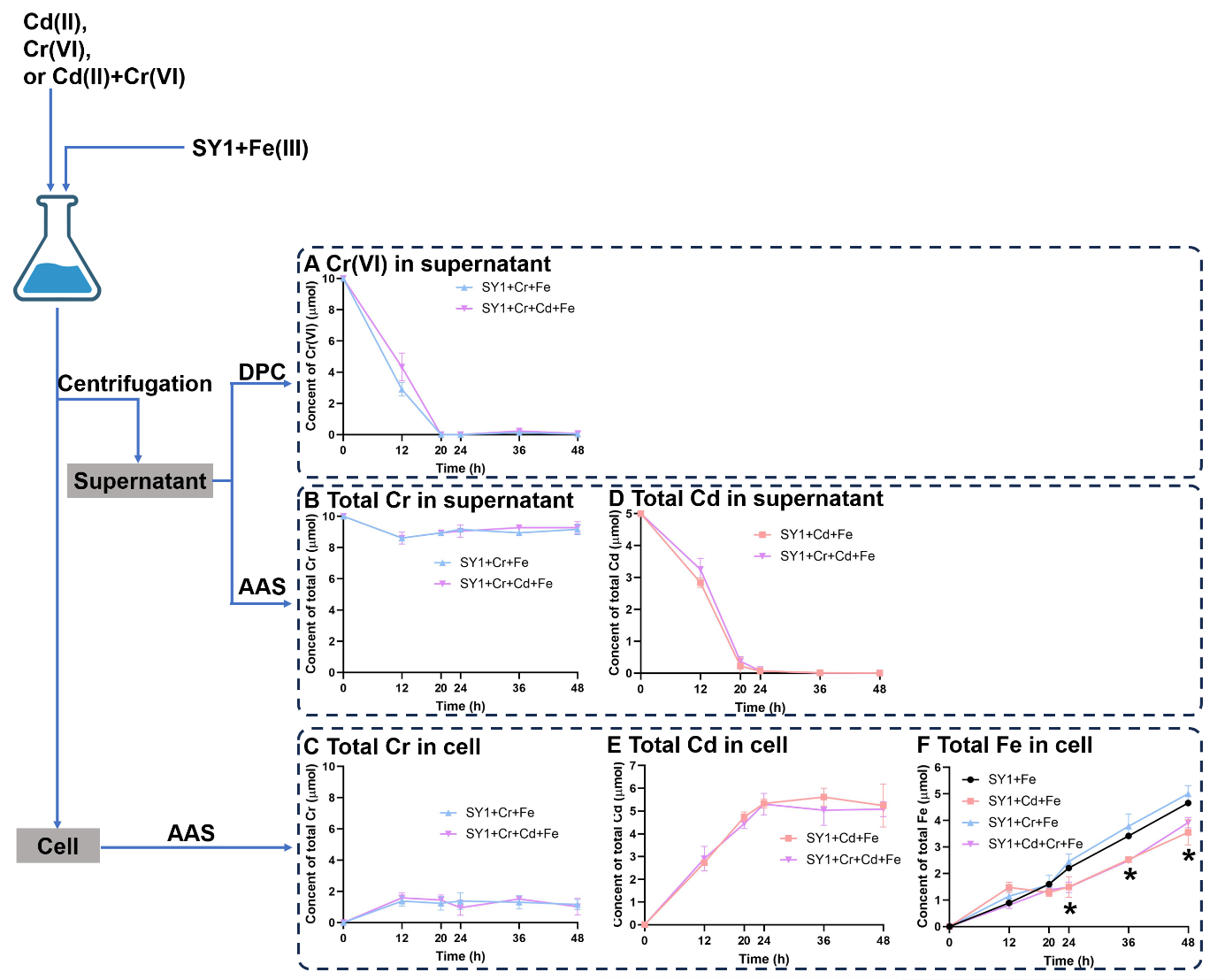


**Figure S9. Cd(II) adsorption and Cr(VI)** **reduction of *Stenotrophomonas* sp. SY1 with the addition of Fe(III).** The strain SY1 was cultivated in LB medium containing Fe(III) with no heavy metal, Cd(II), Cr(VI), or Cd(II) + Cr(VI). **(A)** The content of Cr(VI) in the supernatant was determined using DPC. **(B)** The total amount of Cr in the supernatant was measured by AAS. **(C)** The total content of Cr within the cells was quantified using AAS. **(D)** The total concentration of Cd in the supernatant was analyzed by AAS. **(E)** The cellular accumulation of Cd was determined using AAS. **(F)** The total amount of Fe within the cells was measured by AAS). * indicates a significance difference in Fe content between experimental groups SY1 + Cd + Fe or SY1 + Cd + Cr + Fe and control group SY1 + Fe at each sampling time point (p < 0.05). These data represent average values obtained from three biological replicates.


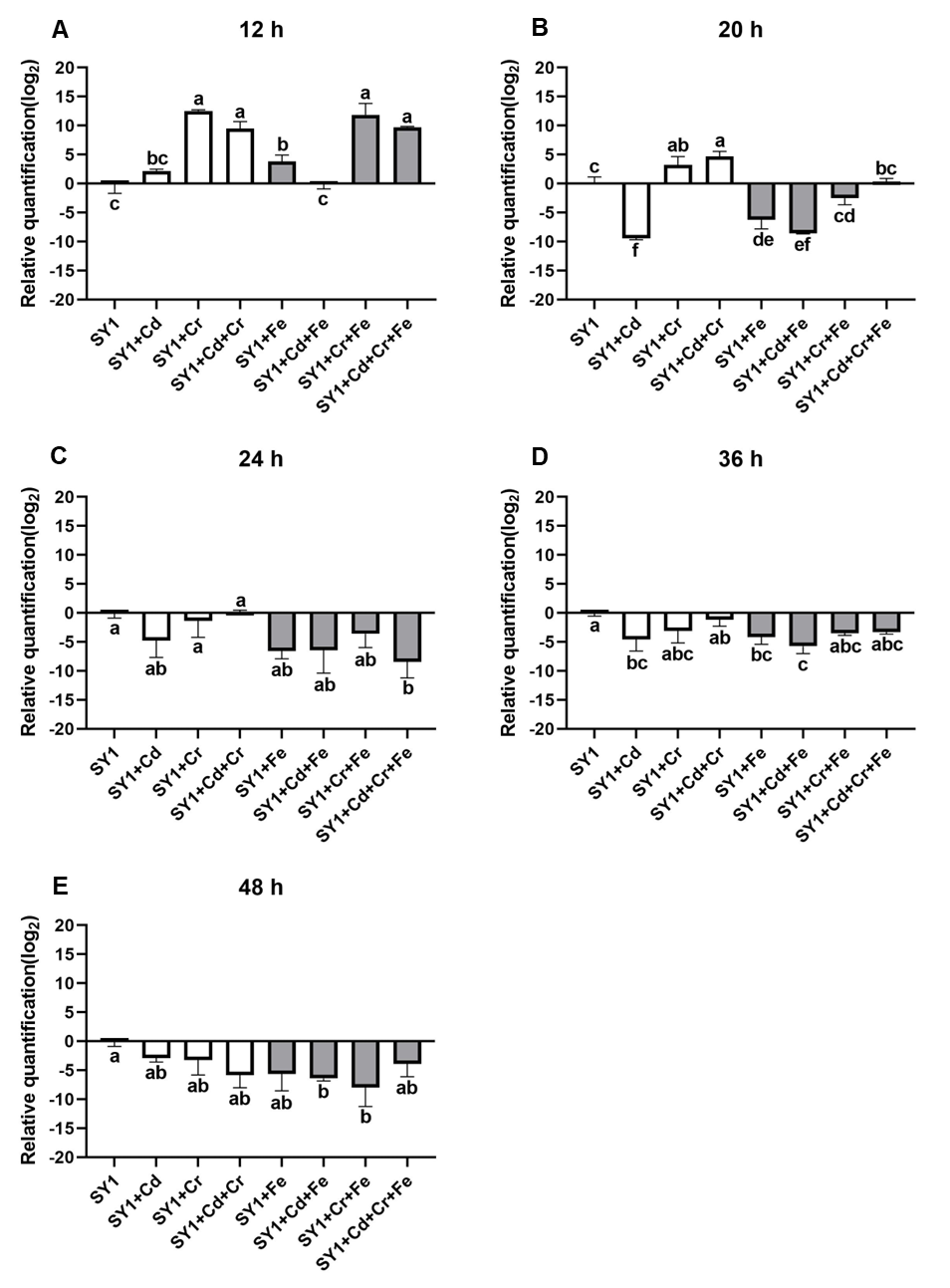


**Figure S10. The gene expression of *hhuH* in strain SY1 with different conditions.** The RT-qPCR analysis of the *hhuH* gene was conducted on strain SY1 under various cultivation conditions at time point 12 (**A**), 20 (**B**), 24 (**C**), 36 (**D**), and 48 h (**E**). The presented data represents the mean values obtained from three biological replicates, with different lowercase letters (abcdef) indicating statistically significant differences between these experimental groups (p < 0.05).


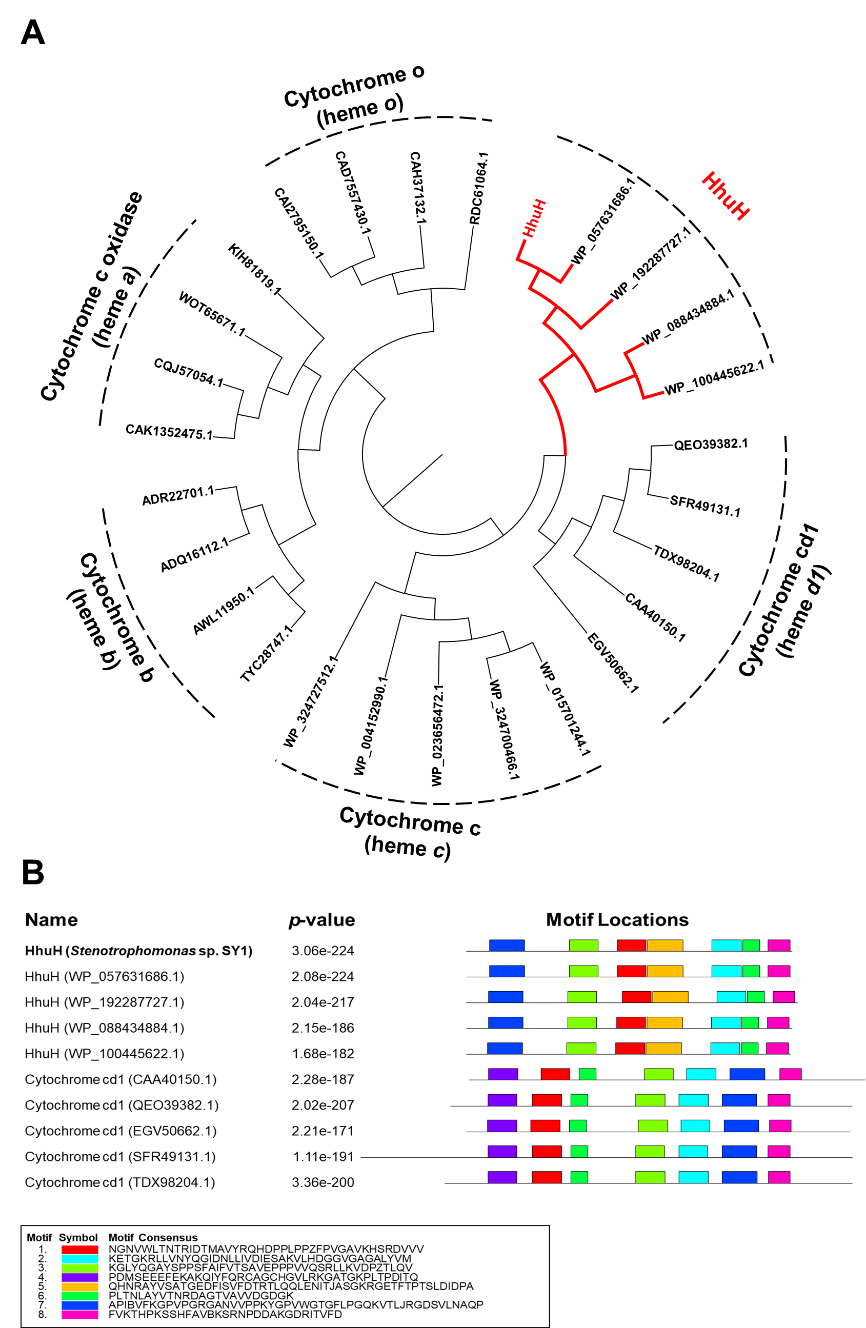


**Figure S11. Amino acid sequences analysis of HhuH and other hemeproteins. (A)** The phylogenetic tree based on the amino acid sequences of HhuH and other hemeproteins, which was performed by MEGA 6.0 with NJ method.. **(B)** The difference of conserved motif between HhuH and cytochrome cd1.


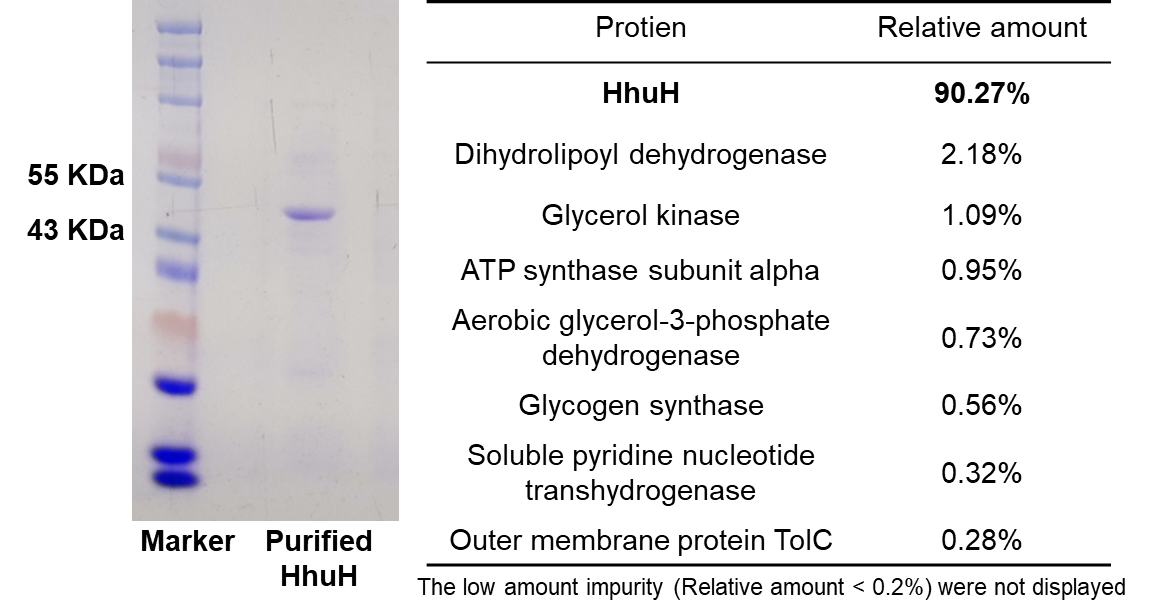


**Figure S12. Identification of purified HhuH protein.** The left figure represents the gel image of SDS-PAGE for purified HhuH, while the right figure corresponds the LC-MS result obtained from purified HhuH.


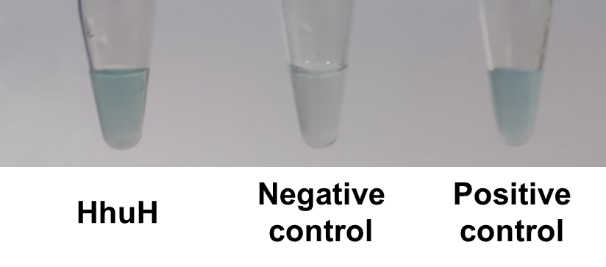


**Figure S13. The ABTS measurement for purified HhuH.** Bovine serum albumin was used as the negative control, while standard hemoglobin served as the positive control.


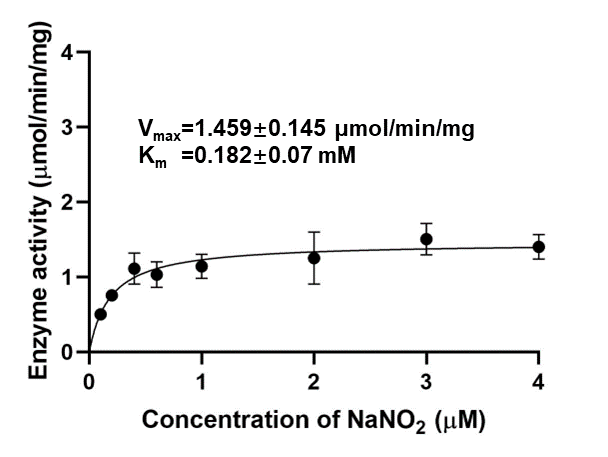


**Figure S14. The enzyme activity curve of HhuH catalyzes the reduction of NaNO_2_.** The data represent the average values obtained from three biological replicates.


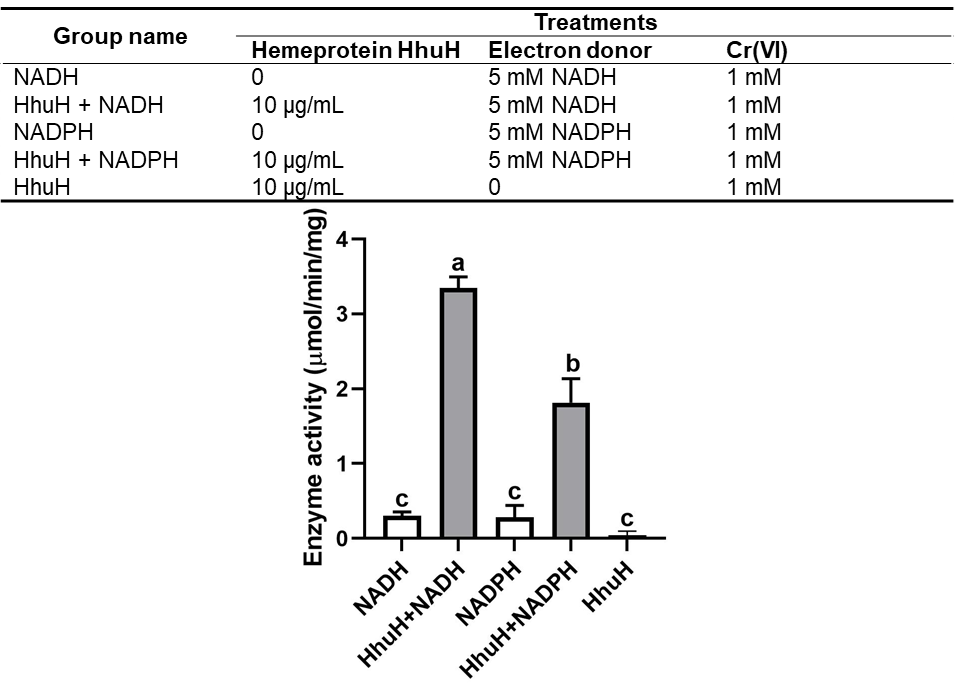


**Figure S15. The non-enzymatic negative control and background enzyme activity of HhuH.** The groups NADH and NADPH were the non-enzyme negative controls of groups HhuH + NADH and HhuH + NADPH, respectively. The group HhuH was the non-electron donor controls of groups HhuH + NADH and HhuH + NADPH. The data represent the average values obtained from three biological replicates, and different letters (abc) indicate significant differences between these groups (p < 0.05).

**
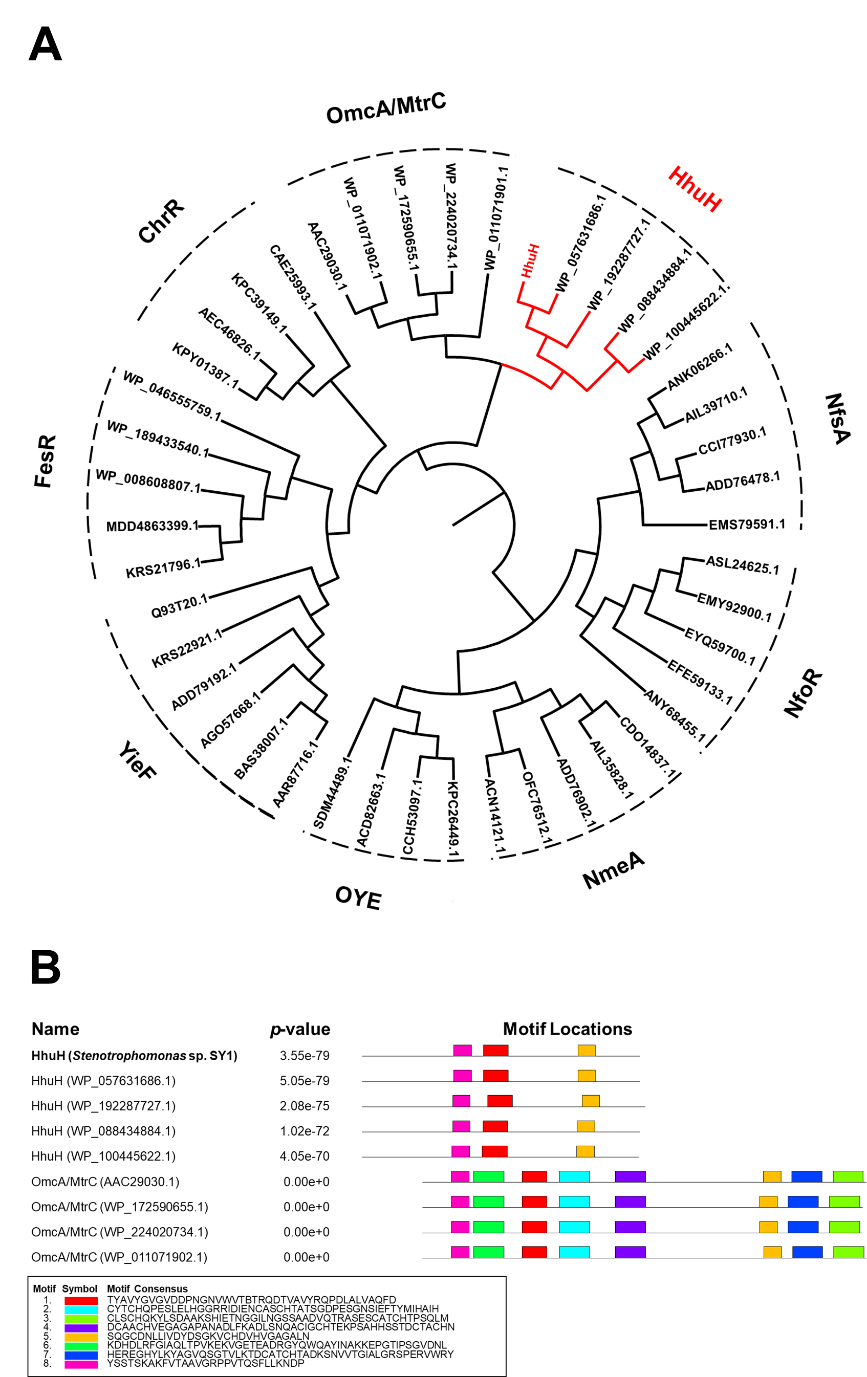
**

**Figure S16. Amino acid sequences analysis of HhuH and other Cr(VI) reductases. (A)** The phylogenetic tree based on the amino acid sequences of HhuH and other Cr(VI) reductases, which was performed by MEGA 6.0 with NJ method. **(B)** The difference of conserved motif between HhuH and OmcA/MtrC.
